# The effect of worked material hardness on stone tool wear

**DOI:** 10.1371/journal.pone.0276166

**Published:** 2022-10-20

**Authors:** Alice Rodriguez, Kaushik Yanamandra, Lukasz Witek, Zhong Wang, Rakesh K. Behera, Radu Iovita

**Affiliations:** 1 Anthrotopography Laboratory, Center for the Study of Human Origins, Department of Anthropology, New York University, New York, New York, United States of America; 2 Composite Materials and Mechanics Laboratory, Mechanical and Aerospace Engineering Department, New York University, Tandon School of Engineering, Brooklyn, New York, United States of America; 3 Department of Biomaterials and Biomimetics, New York University College of Dentistry, New York, New York, United States of America; 4 Department of Biomedical Engineering, New York University Tandon School of Engineering, New York, New York, United States of America; 5 Department of Early Prehistory and Quaternary Ecology, Eberhard Karls University of Tübingen, Tübingen, Germany; University of Vigo, SPAIN

## Abstract

The identification of ancient worked materials is one of the fundamental goals of lithic use wear analysis and one of the most important parts of understanding how stone tools were used in the past. Given the documented overlaps in wear patterns generated by different materials, it is imperative to understand how individual materials’ mechanical properties might influence wear formation. Because isolating physical parameters and measuring their change is necessary for such an endeavor, controlled (rather than replicative) experiments combined with objective measurements of surface topography are necessary to better grasp how surface modifications formed on stone tools. Therefore, we used a tribometer to wear natural flint surfaces against five materials (bone, antler, beech wood, spruce wood, and ivory) under the same force, and speed, over one, three, and five hours. The study aimed to test if there is a correlation between surface modifications and the hardness of the worked material. We measured each raw material’s hardness using a nano-indentation test, and we compared the surface texture of the flint bits using a 3D optical profilometer. The interfacial detritus powder was analyzed with a scanning electron microscope to look for abraded flint particles. We demonstrate that, contrary to expectation, softer materials, such as wood, create a smoother surface than hard ones, such as ivory.

## Introduction

Along with the study of ancient residues, microscopic use wear analysis (MWA) is one of the two major and complementary methods of lithic traceology [1], the science of forensically interpreting ancient stone tool use. Despite its immense interpretive potential, its early spectacular results [e.g., 2] were tempered by the blind testing crisis of the 1980s [3, 4], which put into question different researchers’ ability to identify the same worked materials. Despite many advances since the 1980s [see 5–8 for reviews], the identification of different worked materials is still considered an insufficiently developed area. This is a major setback for prehistorians, because residues are not always preserved, and knowing which materials were worked can make an enormous difference in interpreting area or site function, or in claims about style and cultural transmission. Among use-wear micro-traces, researchers often refer to polish as glossy areas observable on stone tools and related to these uses. Polish has been found useful in identifying and distinguishing the worked material [2, 9–14].

The problem is manifold: not all polishes are equally easy to see and interpret, and not all types of data deliver the same answers. Some types of materials, such as cereals [15, 16] or antler, bone, and ivory (ABI) have been identified consistently in blind tests or have been separated quantitatively in experimental settings with varying degrees of success (see [9–11, 17, 18]). Traces left by others, especially by soft and elastic materials, such as meat, tendons, etc., are less identifiable [19]. Profilometers, instruments used to extract micro-surface texture of samples, give the possibility to quantify polish formed on stone tools. Because profilometers measure the texture of the surface and not its glossiness, it is more correct to use “surface modifications” instead of polish when describing samples analyzed with profilometers, therefore this is the term we chose to use in this paper. Several quantitative studies conducted with profilometers did report success in distinguishing target materials, but, even here, within-group similarities were not adequately explained. For example, Stevens et al.’s [10] discriminant functions grouped surface modifications caused by antler-working and plant-working. Moreover, some studies could not clearly distinguish surface texture modifications caused by wood-working [20] and hide-working [13] from unworked areas. In one of the most recent studies, Ibáñez et al. [17] report a similar overlap between some contact materials in their quantitative analysis to those noted by both other quantitative studies and also in blind tests [e.g., 21], despite studying one of the largest samples to date. We cannot understand why attempts to classify surface modifications produced by different worked materials fail or produce ambiguous results because the processes that lead to these differences and similarities in surface modifications are not sufficiently well understood.

One obvious way to resolve this problem is to study the mechanical properties of prehistoric target (worked) materials and test their effects on wear development. Unfortunately, these materials are not commonly studied by scientists and engineers who study wear processes (tribologists), as they have no industrial applications. They are also more variable than industrial ones, as the available data show. For example, the varying amount of mineralization in bone, ivory, and antler causes mechanical properties to also vary among species [22], in some cases significantly so [23]). Further, these materials change their properties when they are wet vs. dry [24], or in very cold weather conditions [particularly ivory, see 25]. While some have claimed that this produces different traces on stone tools, *why* that might happen remains insufficiently explained.

Visible wear traces on stone tool surfaces are currently thought to result from the abrasion of the natural roughness peaks [26–28]. In Schmidt et al. (2020) [28], we recently demonstrated that the main competing model, which asserts that polish is a deposited layer formed by a chemical reaction between the stone surface and the worked material tested in this experiment [29–31], is not correct. Given that the literature abounds in mentions of hardness as a way to group materials, it makes sense to test first the effect of hardness [e.g. 4, 10, 11, 32, 33]. Until now, systematic mechanical tests of archaeologically-relevant target materials have not been incorporated into lithic use-wear research. In particular, hardness tests were carried out on the stones themselves [34–37], but not on the target materials. For this reason, we decided to carry out a test of the target materials’ hardnesses and to evaluate their role in abrading flint with the expectation that the harder the worked material is the more surface modifications will develop on flint.

## Method

### Experimental setup

#### Experiment principle

**Fig 1:**
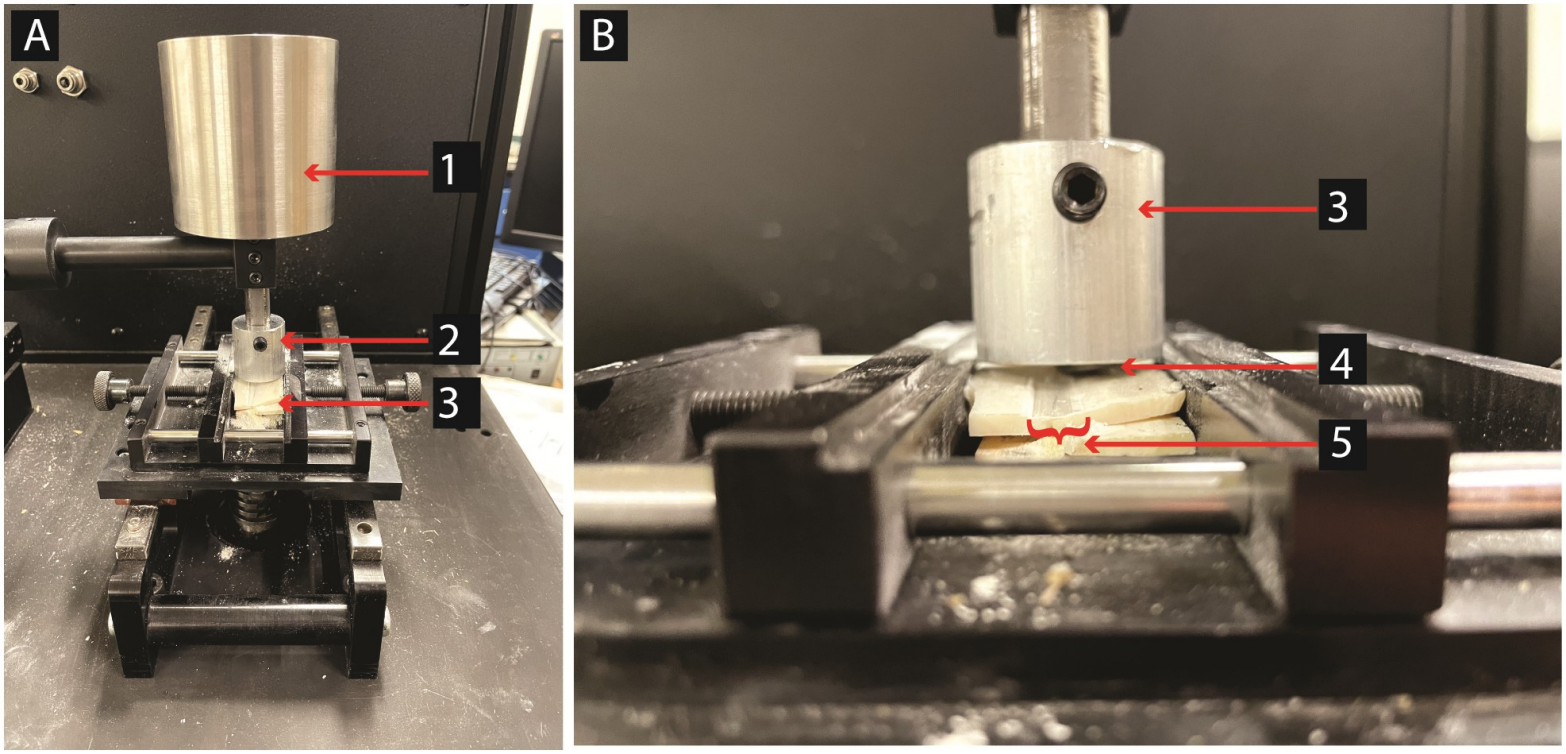
Tribometer setup. A: overview; 1: Load(20N); 2: sample holder; 3: worked material (ivory). B: close-up; 3: worked material (ivory); 4: chert bit; 5: wear track.

To isolate the effect of target materials hardness while maintaining reasonable costs and effort, we opted for a tightly controlled protocol [38, 39]. Flint bits (Baltic/morainic flint from Denmark with nano-crystalline non-oriented chalcedony texture [28]) were rubbed against dry bone (cow bone (*Bos*)), antler (deer (*Cervidae*)), ivory (African Elephant ivory donated to NYU from customs at JFK Airport), and wood (beech and spruce, store-bought) using a tribometer at room temperature. The study included a total of eight (8) flint samples (one sample worked on beech wood, one sample worked on spruce wood, two samples worked on ivory, two samples worked on antler, and two samples worked on bone). The number of flint samples is quite small. However, given the tight controls this is sufficient, because 3–5 surface measurements were taken for each stage of abrasion. The experiment was sequential: the flint samples were mounted on the tribometer (Nanovea T-50) (Fig 1) and used for one, three, and five hours. The tribometer variables were fixed to a load of 20N, a speed of 35 revolutions per minute and a straight back and forth motion.

#### General working principle of the tribometer

**Fig 2:**
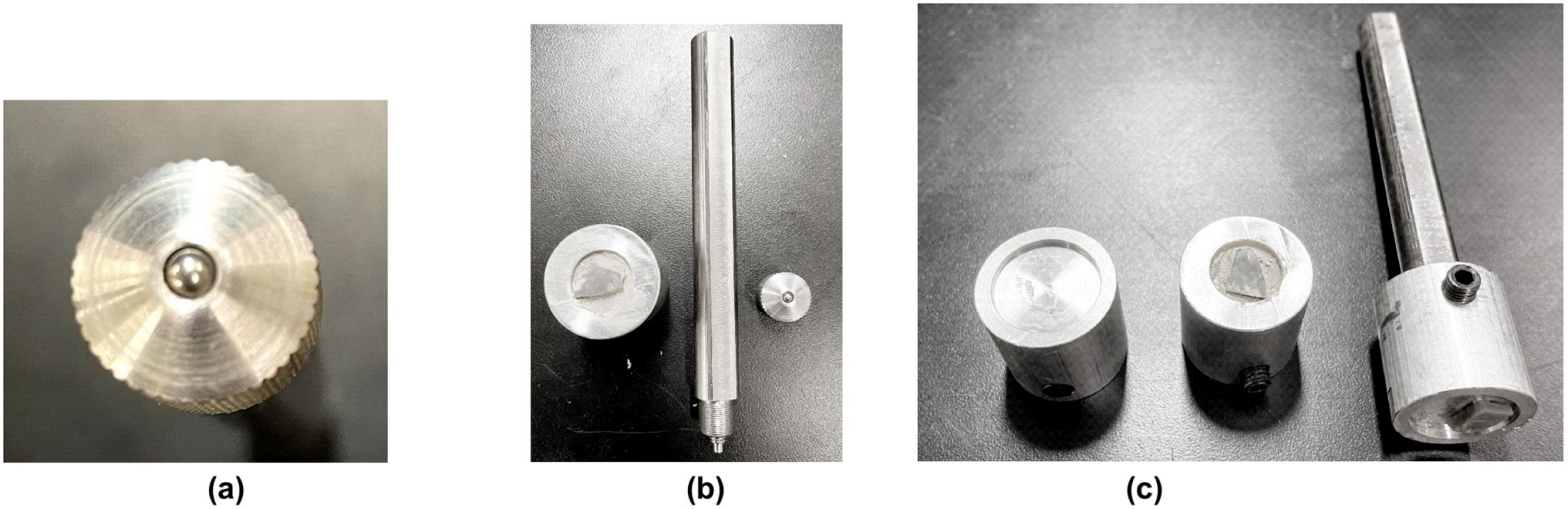
Pin holder types. (a) Conventional Steel ball used in pin-on-disk type of Tribology Experiment, (b) Customized Sample holder setup (on left) conventional sample holder (on right), (c) Customized sample holder, Customized sample holder with Flint sample & the stone sample on the sample holder rod (from left to right).

Tribometers help replicate real-life applications in a wide range of industries including Automotive, Aerospace, Consumer Products, and Industrial & Research Applications. Robust Tribometers provide highly accurate and repeatable wear and friction testing compliant with ISO and ASTM standards. This is achieved by applying a constant load on the material to be worked through a Pin, Ball, or sample by continuous movement generated by rotation or linear to and fro motion. Depending on the environment in which one would like to use the specimen, the tribometer can be equipped with accessories to test the samples in various environments like liquid, high temperature, or humidity conditions. Our instrument performs all the experiments at atmospheric conditions. To perform the archaeological experiments, we have customized the sample holder, which gave us the flexibility to use flint samples for the wear analysis (Fig 2). A linear reciprocating stage, where the rotating motion of the spindle is converted to linear to and fro motion to mimic the tribological motion of cutting, was used for all our tribology experiments. After performing various controlled experiments under different loads, we set the normal load to be 20 N for tribological experiments. The reciprocating stage moved at a speed of 50 RPM (3000 strokes per hour) and an amplitude of 20 mm. We decided on 20N because it was the highest load we could use to maintain a smooth motion. We performed the experiments at three different durations (one hour, three hours, and five hours) to understand the development of wear on the different combinations of flint and worked materials (Table 1). Each experiment collects the information of wear with respect to the reciprocating motion or time as well as the force vs. coefficient of friction during the test. In experiments where humans conduct activities with stone tools, the surface modification can appear as fast as 10 minutes. However, this setup uses a load smaller than humans can produce. Hence a longer working time was necessary to obtain a modified surface. We evaluated that one to five hours of work were required to obtain texture modifications on the stone surface with this setup.

**Table 1:** Input parameter used for the tribological experiment with the archaeological samples.

Instrument	Nanovea T50		
**Normal Force (N)**	20N		
**Rotative Speed (RPM)**	50		
**Duration of Tests**	1hr	3hr	5hr
**Number of Strokes**	3000	9000	15000

#### Sensors and calibration

**Fig 3:**
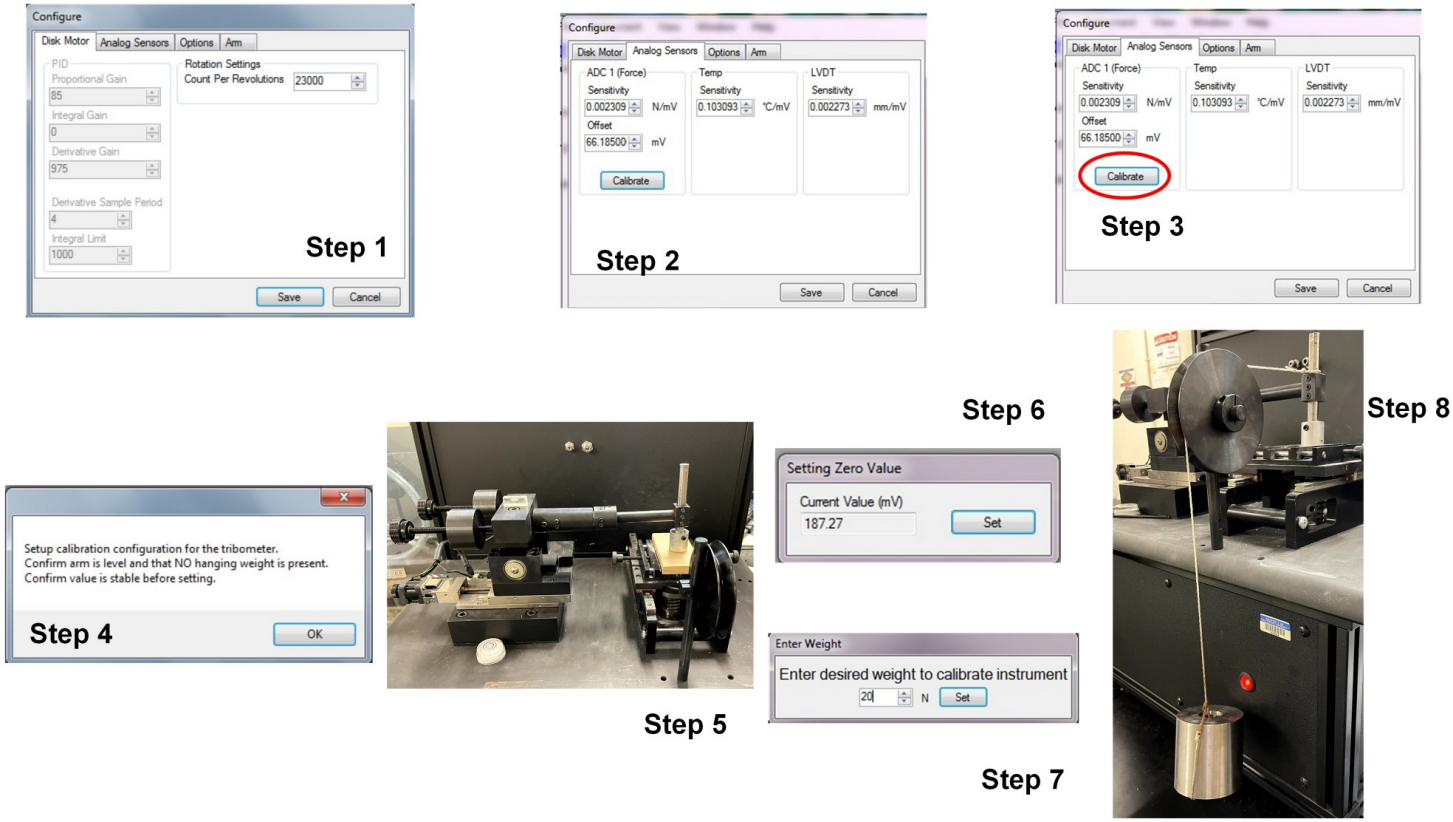
Step by step procedure for calibration of the load sensor in Nanovea T50.

Nanovea T50 has various sensors to provide accurate load and wear information. In the T50 tribometer used in this paper, we have a LVDT/depth sensor and a friction sensor (precision load cell). For calibration of T50, we have two different parts (Disk Motor and Analog Sensors) where user input is needed before performing any experiment for data collection. Disk Motor is generally not calibrated unless the software sends a prompt to calibrate. Therefore, our focus mainly was to calibrate the Analog Sensors (primarily the load sensor). A step-by-step procedure to calibrate the load cell is given in Fig 3. Steps 1–4 are controlled by the software interface where we prepare the instrument for calibration with no hanging weights on the tribometer arm. Once we get to step 5, we raise the tribometer arm. In the following step, we set the load value to be zero. In step 7, we enter the calibration load necessary for the experiment. For our case, we set 20 N as the calibration load. In Step 8, we place the 20 N load on the calibration lanyard and place the other end over the sample holder as shown. It is important to verify that the lanyard is horizontal between the pulley and the sample holder. Once the calibration weight stops swinging, we set the load to complete the calibration. The instrument was calibrated before every run on the archaeological samples.

### Sample preparation

#### Flint bits

The stone was first broken into small fragments using a hammer. The flint bits that had a flat natural surface on one side were selected. The opposite side was then adjusted to fit into the tribometer’s clamp using a saw (Buehler, ISOMET TM, low speed saw) and then glued (Scotch Create Permanent Super Glue Liquid) to the clamp. The flat natural surface was rubbed against one of the worked materials selected. As surface modifications appear unevenly on the sides of the sharp edge of a tool, this setup helps to create a sufficiently large modified area for carrying out microscopic analyses. Samples were manipulated with gloves and were cleaned before every documentation of wear, once to document the surface roughness before use and once after each tribological sequence. Because of the metallic nature of the clamp, a harsh cleaning protocol using acid and base was impossible and, instead, a mild cleaning process was used. The samples were placed in individual plastic bags filled with a 10% neutral soap solution (Valconox, Luminox). They were then immersed into an ultrasonic bath for 15 minutes (Branson 5800, 40 kHz, at room temperature, 22°C). Then, they were rinsed with tap water and put in a distilled water bath for 5 minutes. Finally, samples were left to air-dry. Reflectance infrared spectroscopy was performed on both used and unused surfaces of the flint bits. Results were presented in a previous study [28]. Both surfaces are composed of quartz (crystalline silica), the main component of flint. These results confirmed that the mild cleaning method used during the experiment successfully removed residue from the flint bits before they were observed under a microscope.

#### Hardness tests for flint and target materials

The hardness of each raw material (flint and worked materials) was measured using a nano-indentation test. The samples were placed in resin using a SamplKwick Fast Cure Acrylic Kit 20–3560 (which contains SamplKwick Powder 20–3562 and SamplKwick Liquid 20–3564). The resin was prepared by a cold molding process done at room temperature by mixing two parts of 20–3562 SamplKwick Powder and one part of 20–3564 SamplKwick and blending thoroughly for 15–20 seconds. Then the mixture was poured into ring molds without delay. Before pouring the mix into the ring molds, we sprayed the cups with mold release spray (Buehler Mold Release Spray, 203050008). Finally, the surface of the cast was polished to obtain a smooth surface.

**Fig 4:**
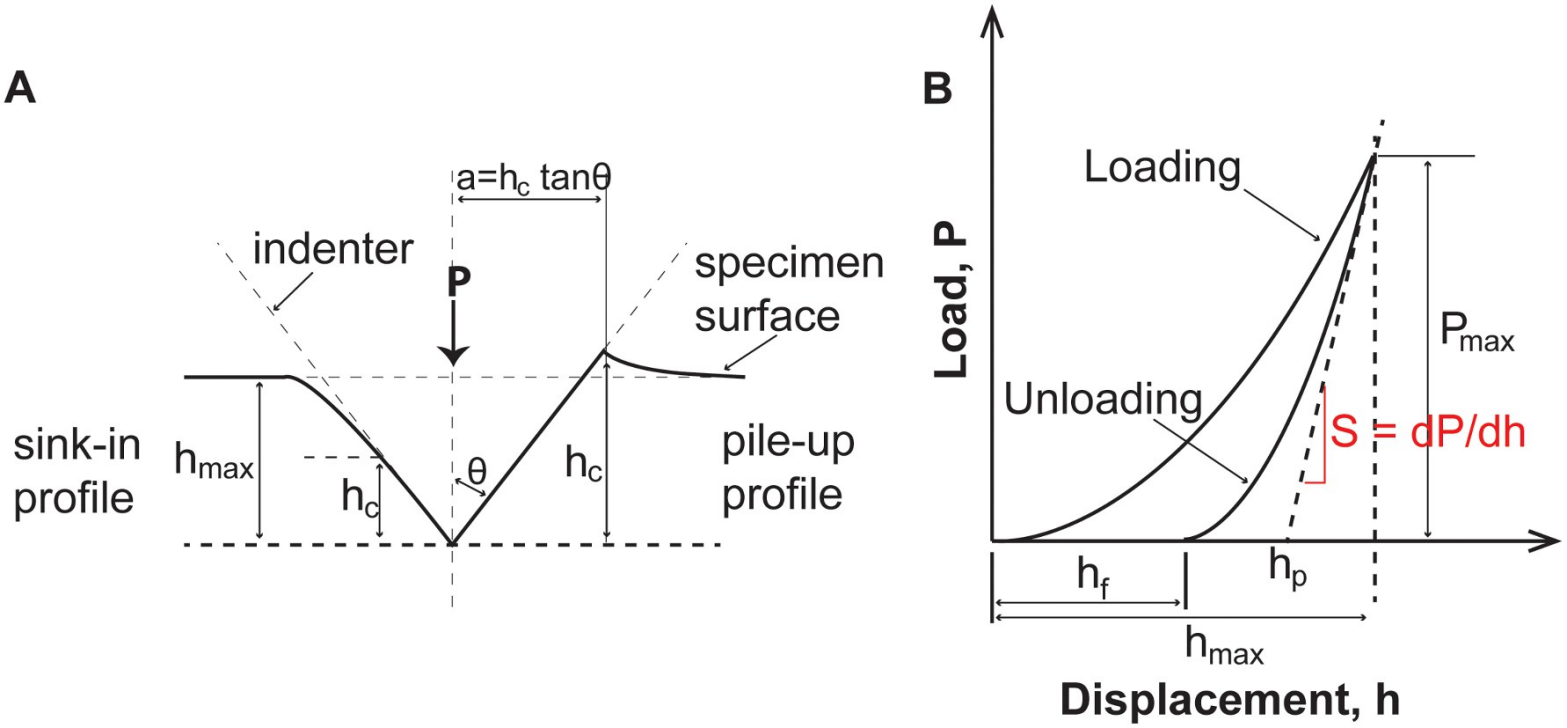
Material reaction during the nanoindentation process. A: Schematic drawing showing the surface displacement during indentation; B: A typical indentation P-h curve where h_c_ is the maximum true contact displacement during loading, P_max_ refers to the maximum load applied during the indentation cycle, S is the initial unloading stiffness, h_f_ is the final plastic depth from the P-h curves, h_s_ is the residual depth after complete unloading and h_max_ is the indent.

The nanoindentation measurements were performed using a nanoindenter (TI 950, Triboindenter, Hysitron, Minneapolis, MN) equipped with a diamond Berkovitch indenter. Prior to the experiment, the tip area function and the frame stiffness were calibrated using a fused silica standard. Nanoindentation measurements were conducted using the Oliver-Pharr or the quasi-static loading mode [40–42] from which the reduced modulus (*E*_*r*_) and hardness were estimated (Fig 4) The initial unloading portion of the load-displacement curve represents purely elastic recovery. The slope of this unloading segment is a measure of the material contact stiffness [43]. The reduced Young’s modulus (*E*_*r*_) can be calculated by

$$E_{r}=\;\frac{1}{2}\;S\;\sqrt{\frac{\pi }{A_{max}}}$$ (1)

where S is the contact stiffness and *A*_*max*_ is the surface contact area at the maximum depth. The elastic modulus of the indented specimen, *E*_*S*_ is computed using

$$\frac{1}{E_{r}}=\;\frac{1-\upsilon_{S}^{2}}{E_{S}}+\frac{1-\upsilon_{i}^{2}}{E_{i}}$$ (2)

where *υ*_s_ is the Poisson’s ratio of the indented specimen and E_*i*_ and *υ*_*i*_ are the Young’s modulus and Poisson’s ratio, respectively, of the indenter. For a diamond Berkovitch indenter, *E*_i_ is equal to 1141 GPa and *υ*_*i*_ is equal to 0.07 [44]. Since the Poisson’s ratio of sedimentary rock ranges between 0.1–0.3, we have decided to report the *E*_*r*_ rather than estimate the *E*_*S*_ for modulus comparison (see S1 Appendix) [45].

In addition, the hardness (*H*) can be calculated using the maximum load, *P*_max_, by

$$H=P_{max}/A_{c}$$ (3)

where *A*_*c*_ is the contact area of the indentation. These quasi-static measurements were performed with a 5 s load time and a 10 s dwell time at a maximum load of 3000 μN for stones and 300 μN for, spruce wood, beech wood, bone, ivory, and antler (Table 2).

**Table 2:** Raw materials average hardness and Er values. These values are calculated by taking all the measurements into account for each sample. Materials are listed in increasing order of hardness, from the softest (Spruce) to the hardest (Flint).

	Spruce wood	Beech wood	Bone	Antler	Ivory	Flint
**Reduced Modulus (E_r_ in GPa)**	1.078 ± 0.359	19.307 ± 8.121	21.791 ± 1.172	30.562 ± 5.128	36.439 ± 0.323	49.729 ± 3.515
**Hardness (H in GPa)**	0.122 ± 0.004	2.833 ± 1.672	2.961 ± 0.246	3.253 ± 0.727	3.930± 0.025	6.280 ± 0.672

### Analysis of altered surfaces

**Fig 5:**
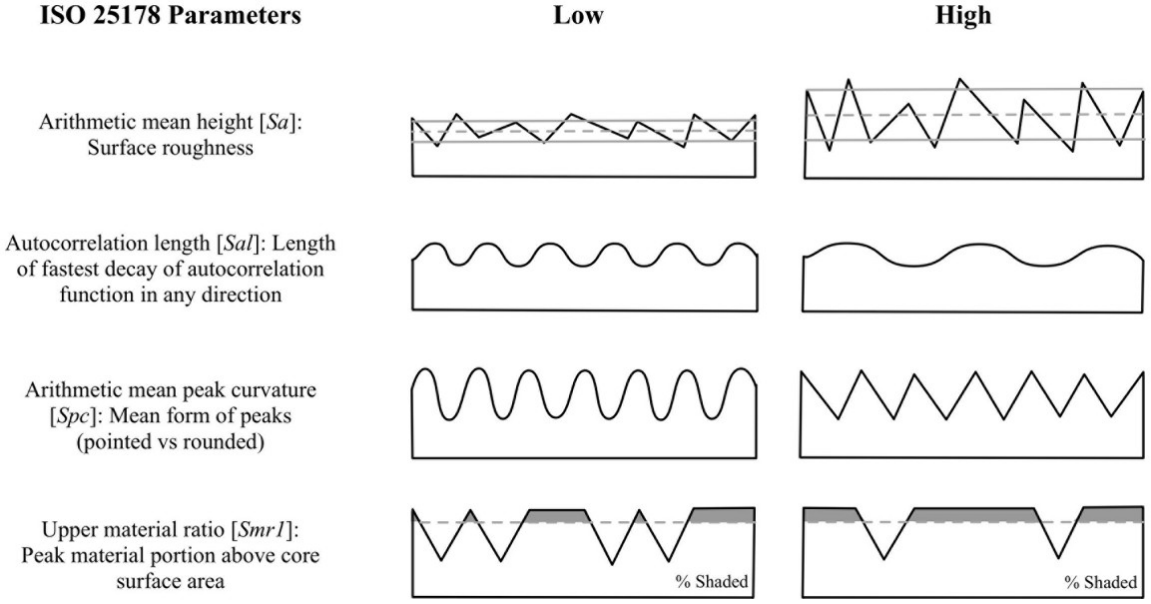
Visual representation of the surface parameters. (from Martisius et al., 2018 [49] (doi: https://doi.org/10.1371/journal.pone.0206078.g004).

An optical profilometer (S-Neox, Sensofar Metrology, Barcelona, Spain) was used to collect surface topography measurements before the experimentation and then at each step of the experiment. At each step, 3 to 5 measurements of different areas were taken for each sample depending on the alteration development. The roughness measures correspond to the whole field of view filled by the altered surface (as identified visually). Measurements were acquired with both a 20× objective (TU Plan Fluor EPI P; NA = 0.45; FoV = 872.68μm × 655.965μm) and a 50× objective (TU Plan Fluor EPI P; NA = 0.80; FoV = 350.88μm × 264.19μm). Pictures were taken using the blue LED (530 nm) to obtain the highest resolution possible with our equipment. Only pictures with a surface measure higher than 98% were used for the analysis. The surface images were then analyzed using SensoMap (Standard 7.4, the equivalent of Mountains Map designed by Digital Surf for Sensofar). To extract surface parameters from the pictures, the filtering protocol presented in Calandra et al. [46] was used: (1) extraction of the topographic layer, (2) use of a Gaussian low-pass S-filter (S1 nesting index = 1.093 μm for the 20× objective and 0.437 μm for the 50×, end effects managed) to remove noise and keep the primary surface, (3) use of an F operator (polynomial of degree 3) to remove the form and keep the SF surface, i.e., texture, (4) use of a Gaussian high-pass L-filter (L nesting index = 327.980 μm for the 20× objective and 131.200 μm for the 50×, end effects managed) to filter out the waviness and keep the SL surface, i.e., roughness, and (5) setting threshold surface between 0.010 and 99.9% material ratio to remove the aberrant positive and negative spikes. It is important to note, however, that sometimes that threshold was readjusted depending on the persistence of the outliers. The cut-off values were calculated following ISO norms [47, 48] recommendations; the L nesting index used was half the size of the shortest side (breadth) of the field of view, and the S1 nesting index was obtained by dividing the L nesting by 300 times. Four of the ISO25178 parameters were selected to perform the statistical analysis (Fig 5): arithmetic mean height (*Sa*), autocorrelation length (*Sal*), arithmetic mean peak curvature (*Spc*), and the upper material ratio (*Smr1*). These four parameters, relatively independent, provide an overall understanding of the surface textures [49]. Moreover, a digital microscope (DinoLite Edge 3.0 AM73515MZT) was used to document the overall bits’ surfaces after five hours of use and pictures of the surface modifications were taken at 100X (TU Plan ELWD; NA = 0.80) with the Sensofar S-NEOX. The residual powders formed by the friction of the stone bit against the worked material were collected and then analyzed (imaging and EDX) using scanning electron microscopy (SEM Hitachi S-3500N).

The statistical analyses were performed in the open-source software R (v. 3.5.2; [50] using the following packages: ggplot2 (v. 3.1.0; [51]), dplyr (v. 0.8.0.1; [52]), tidyr (v. 0.8.3; [53]), ggpubr (v. 0.1.2; [54]), ggalt (v. 0.4.0; [55]), MASS (v. 7.3–53.1; [56]), gridExtra (v.2.3; [57]), cowplot (v. 1.1.1; [58]), rstatix(v. 0.7.0; [59]), and knitr (v. 1.31; [60]). Boxplots were produced to give a sense of the flint texture variation depending on the worked material hardness. Besides, an analysis of variance (ANOVA) and paired t-test were used to show which roughness parameters can help discriminate material types or hardness. Finally, we ran a Kendall’s-tau correlation test (Kendall v. 2.0 [61]) to examine the degree of correlation between the hardness of the worked material and the surface texture of the worked flint bits.

## Results

### Direct observation results

**Fig 6:**
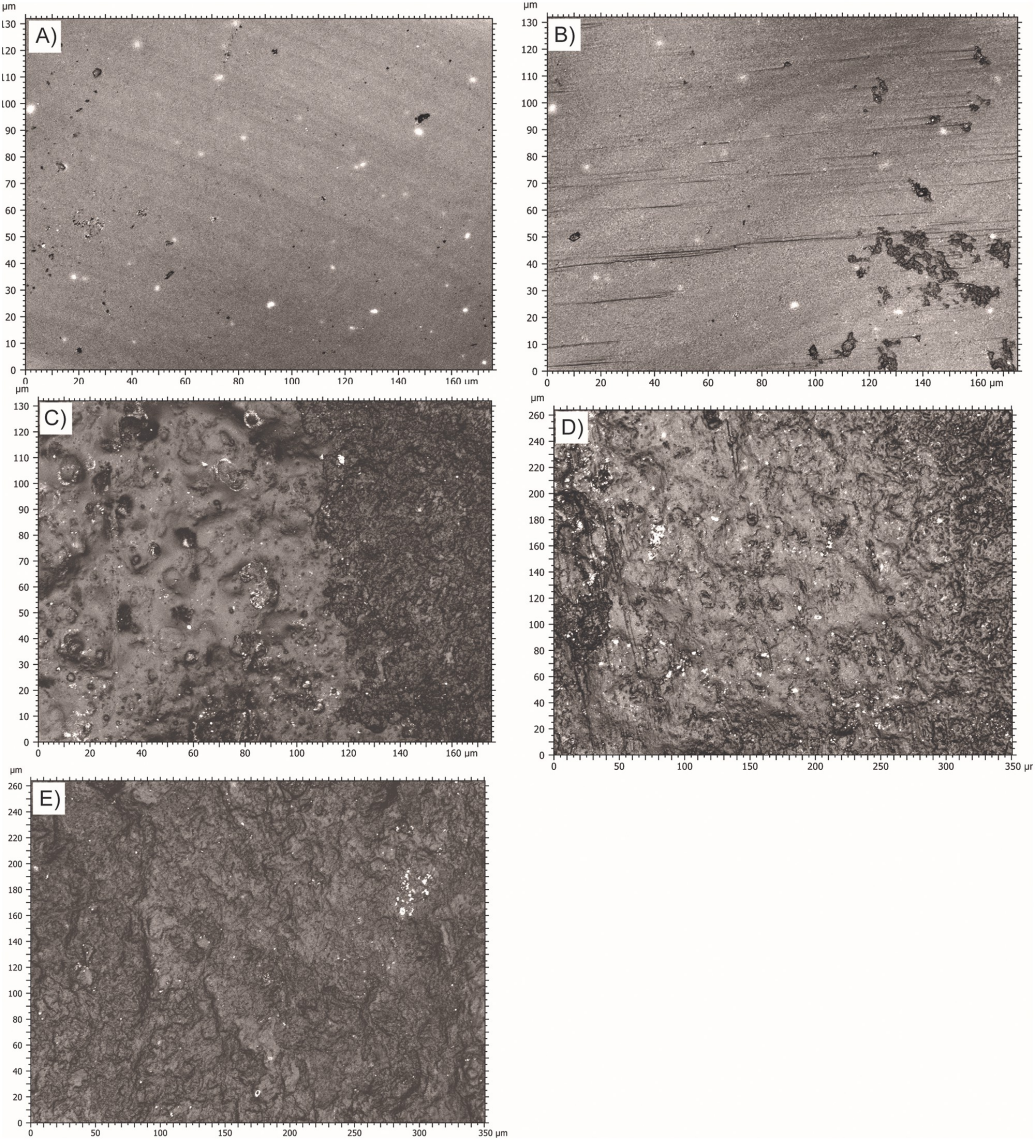
100X pictures of the polished areas taken with the Sensofar. A) Surface modifications formed by rubbing the stone bit on Spruce wood. B) Surface modifications formed by rubbing the stone bit on Beech wood. C) Surface modifications formed by rubbing the stone bit on bone. D) Surface modifications formed by rubbing the stone bit on antler. E) Surface modifications formed by rubbing the stone bit on ivory.

**Fig 7:**
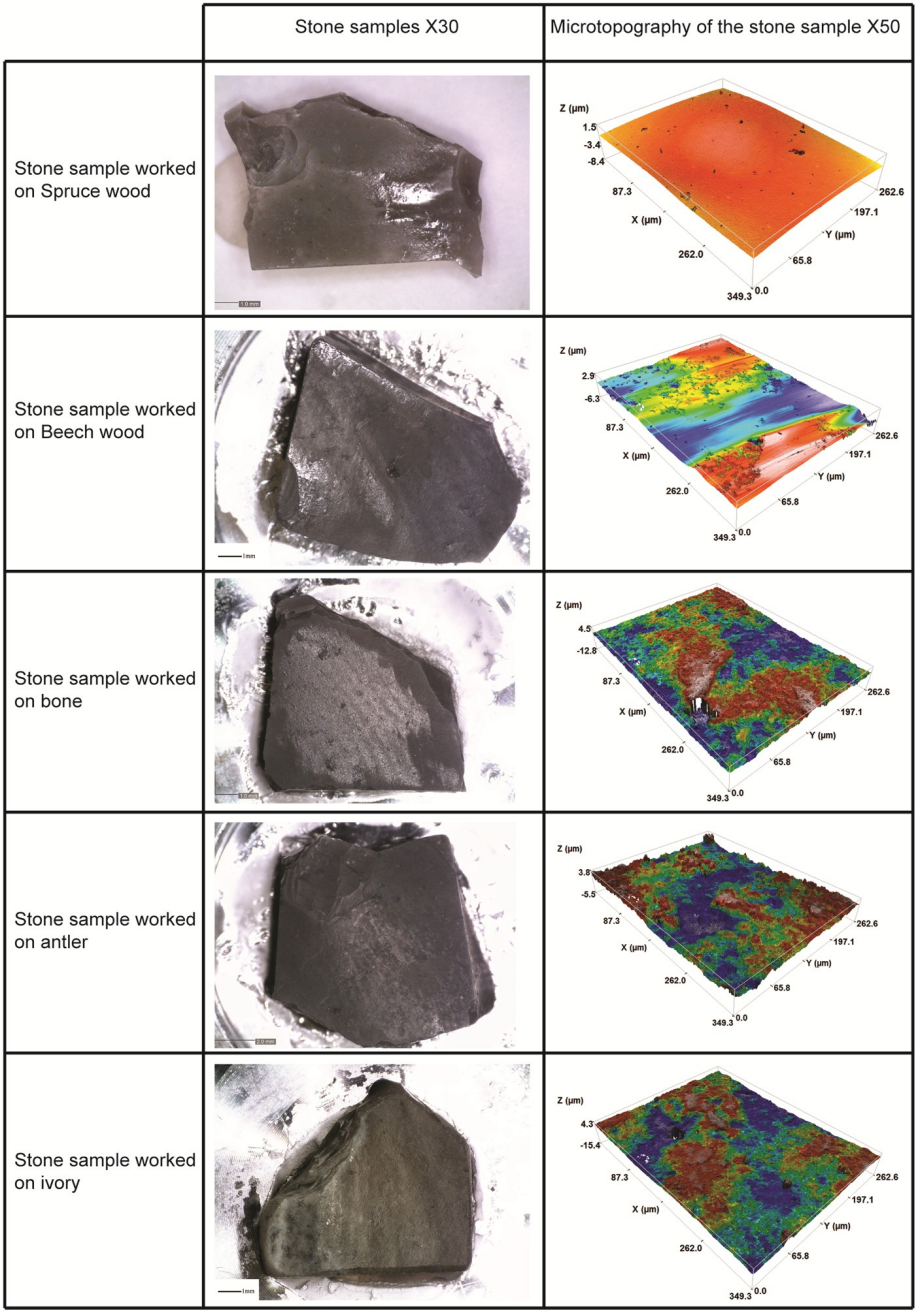
Polish development after 5 hours of use. The samples are organized by hardness order, from the softer on top of table to the harder on the bottom.

Visually, it is noticeable that polish did not develop to the same extent on all the flint bits, as observable at 100X magnification (Fig 6) and even with the naked eye (Fig 7). According to the statistical results, the flint surface appears more polished when rubbed on spruce wood and beech wood. For the other worked material, the polish is sparser.

### Statistical results

**Fig 8:**
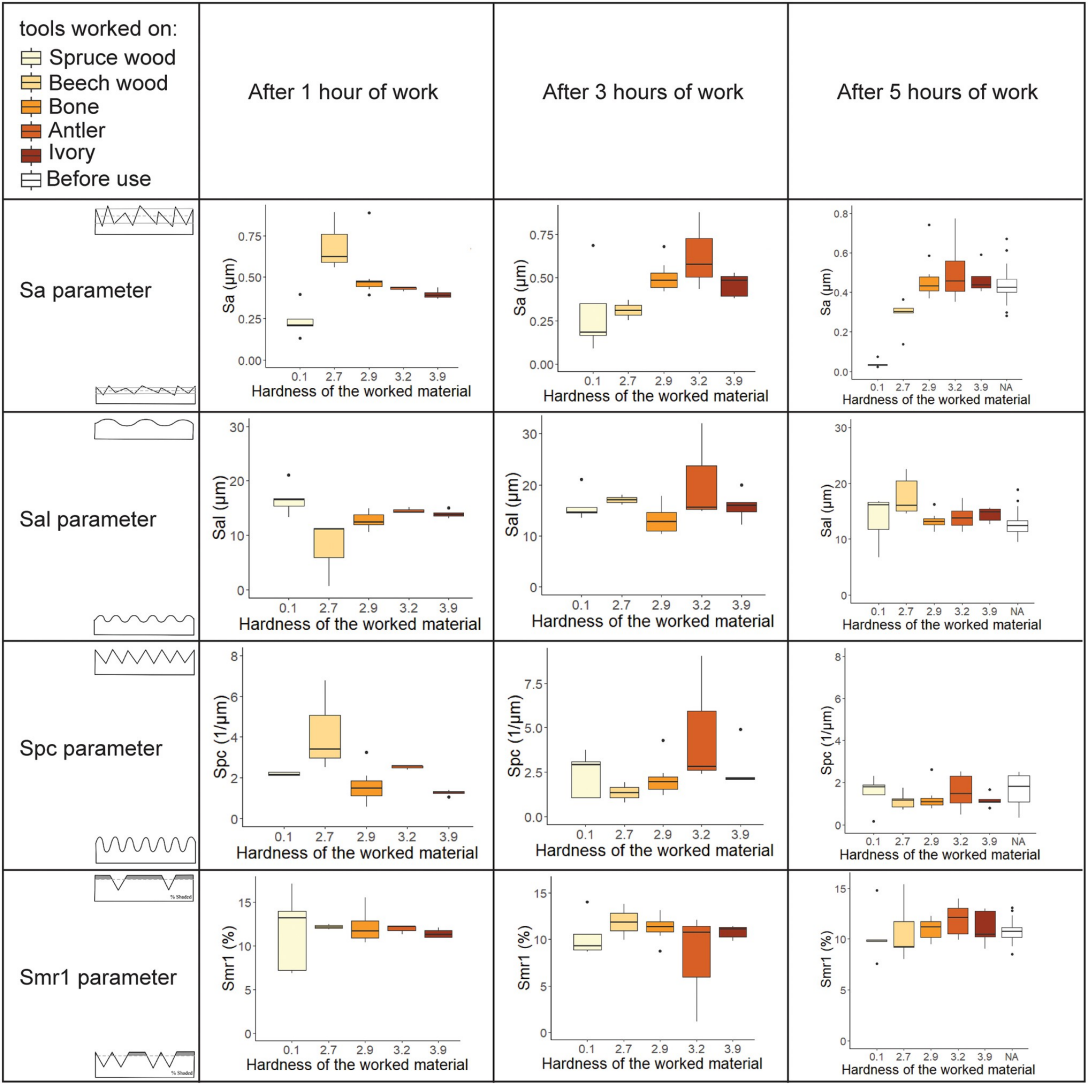
Boxplots showing the evolution of flint roughness parameters Sa, Sal, Spc, and Smr1 after 1 hour, 3 hours and 5 hours of use on worked materials of various hardnesses. The diagrams in the first column represent the expected microtopography for a high value (on the top part of the cell) or low value (on the bottom part of the cell) for each parameter (extract from Martisius et al., 2018 [49]).

The Sa parameter is the only parameter for which the analysis of variance (ANOVA) shows statistically significant difference (p < 0.001) between traces obtained with the different worked material (Fig 8). Hence, *Sa* is the most suited of the four parameters tested for understanding the worked material hardness’s impact on the formation of surface modification. The results indicate that surface modification on flint is influenced by the worked material, and that material hardness plays a role in the development of surface modification. It suggests that softer worked materials will create smoother surface modifications relative to harder materials. Given these results, it is imperative that other mechanical properties of materials typically expected to occur in prehistoric tasks be studied.

Paired t-tests with the Bonferroni correction results for the Sa parameter (see Table 3) show that it is possible to differentiate a flint piece before and after use only for the softest material, the spruce wood (*p* = 0.001). It is also possible to differentiate between the two wood types (*p* = 0.045). However, for antler, ivory, and bone, the paired t-tests show that the means before and after use were not significantly different. In term of duration of the action, the results were statistically significant after 3 hours of use. However, the significance increased after 5 hours of use in particular making it possible to distinguish between the two wood types.

**Table 3:** Paired t-test results with the Bonferroni correction and their significance for the parameter Sa after five hours of use.

Parameter tested	worked materials		number of measurements		statistic	df	p	p-value adjusted using the Bonferroni correction	adjusted p-value significance
Sa	Antler	Beech wood	10	5	3.7703086	11.719557	3.00E-03	0.045	*
Sa	Antler	Bone	10	10	0.5388624	17.567535	5.97E-01	1	ns
Sa	Antler	Ivory	10	5	0.6032733	12.638991	5.57E-01	1	ns
Sa	Antler	Raw	10	40	1.4770012	10.563733	1.69E-01	1	ns
Sa	Antler	Spruce wood	10	5	10.74617	9.772127	1.00E-06	0.0000149	***
Sa	Beechwood	Bone	5	10	-3.5174487	10.386906	5.00E-03	0.075	ns
Sa	Beechwood	Ivory	5	5	-3.5850431	7.819647	7.00E-03	0.105	ns
Sa	Beechwood	Raw	5	40	-3.712811	4.82499	1.50E-02	0.225	ns
Sa	Beechwood	Spruce wood	5	5	6.176421	4.414617	3.00E-03	0.045	*
Sa	Bone	Ivory	10	5	0.0525376	11.680601	9.59E-01	1	ns
Sa	Bone	Raw	10	40	0.9199148	11.167691	3.77E-01	1	ns
Sa	Bone	Spruce wood	10	5	11.684085	10.044221	4.00E-07	0.0000054	***
Sa	Ivory	Raw	5	40	0.910415	5.136519	4.03E-01	1	ns
Sa	Ivory	Spruce wood	5	5	12.455777	4.561824	1.07E-04	0.001605	**
Sa	Raw	Spruce wood	40	5	26.334601	24.570355	0.00E+00	0	***

In the adjusted p-value significance column ns refers to non-significant, and the number of stars to the level of significance. Level of significance:

*p<0.05,

**p<0.001,

***p<0.0001.

The Kendall’s-tau correlation test (Table 4) shows a strong positive statistical relationship between the hardness of the worked material and the modification of the surface texture only for the Sa parameter. This test confirms that the harder the worked material is, the greater the Sa value.

**Table 4:** Result of the Kendall’s tau correlation test for the four texture parameters.

	Kendall’s-tau-B	p-value
**Sa**	0.54	2.79E-05
**Sal**	-0.0922	0.48188
**Smr**	0.228	0.078715
**Spc**	-0.0132	0.92995

### Track analysis

**Fig 9:**
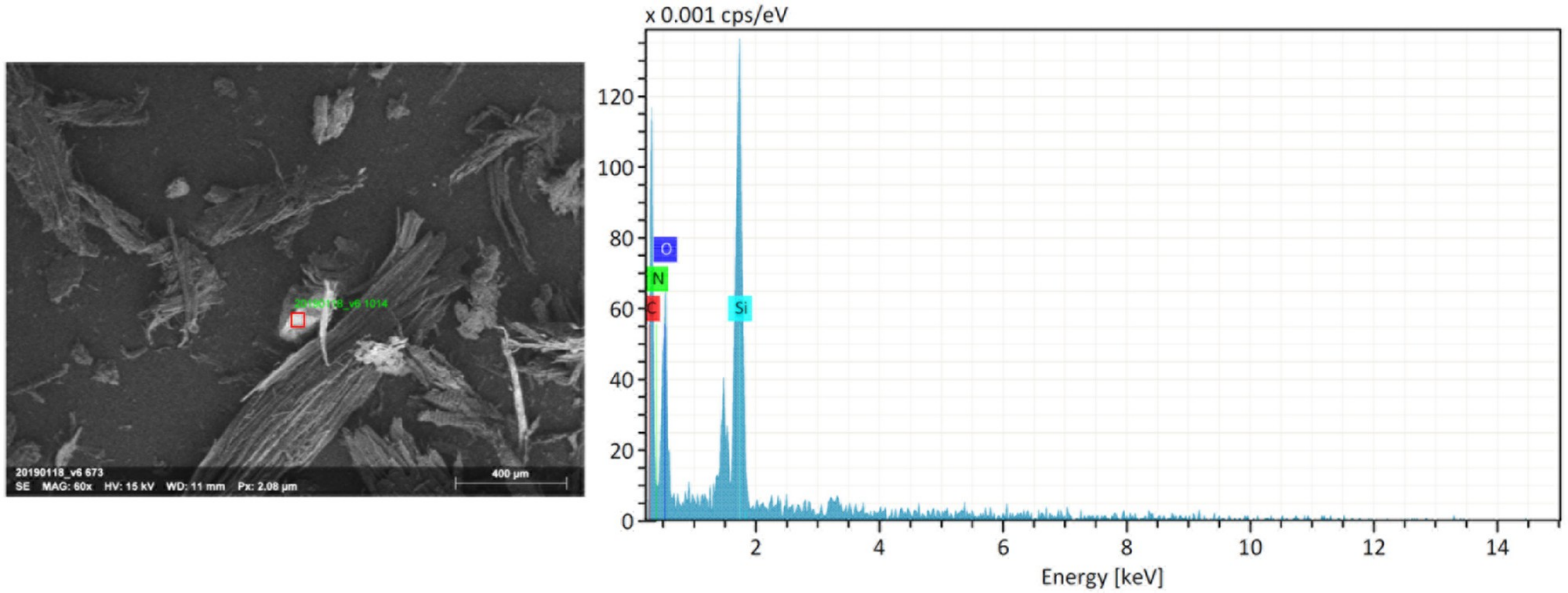
X-ray microanalysis of the spruce wood powder. The red square represents the scanned area. This area is mainly composed of Silicon, suggesting that the fragment (white on the picture) is a flint fragment.

One explanation for these results could be the presence of more abrasive compounds in the softer worked material such as silicate. Residual powders from the track formed by the flint rubbing on the worked materials were collected and analyzed using scanning electron microscopy (Hitachi S-3500N) and X-ray analysis (EDS) (Fig 9). Silica could not be found in the EDS framing the broad region of the samples, most likely because the other elements’ noise was too heavy to enable the detection of other compounds or because the powdered silica are too small to be picked up by the EDS probe. However, we found that the interfacial powders from each raw material contained flint fragments of approximately 200μm.

In this case, the two softer materials happen to be wood, but silica content in temperate wood species is negligible [62], whereas the apatite contained in the bone matrix are softer than the quartz in flint. At the same time, because flint was found in track powders associated with all materials, it is possible that these particles contributed most to the abrasion, since they are hard enough to scratch the stone surface. However, we do not understand exactly how and why flint particles were broken off the stone surface in the first place. Although we tried to maintain a contact between two relatively flat surfaces to maximize contact area and minimize the variation in pressure distribution, because we opted for a natural raw surface, edge breakages were still possible.

## Discussion

The basic problem in explaining what causes stone tool wear is that most worked materials (such as skins, wood, bone, etc.) are softer than stone and cannot, in theory, abrade the stone tools themselves. However, given that this nevertheless happens [26–28, 63], which factors related to the target material play the most significant role? Our counterintuitive result suggests that the abrasion ‘paradox’ must be explained by either the presence of hard grits *within the worked material*, the presence at the interface *of stone particles broken off* the tool edge, or a combination of both [64]. The role of plant silica (phytoliths) in lithic use wear formation is still not completely understood [62], especially with respect to the different quantities present in different wood species [65]. However, we do not have any reason to believe it played a large role in this study, because the track powder did not contain any phytoliths, which would have been sufficiently large to be detected with the SEM. As we found flint particles in every track’s powder, it could be expected that they create homogeneous surface modifications. However, our results show that modifications are not homogeneous and differ depending on the worked material. In addition, the particles found were too big to produce the type of modification observed in particular on the sample with less surface modifications. The last possibility is that still finer particles are removed by contact with the wood via a combination of friction heat and mechanical dislodging. A worked material’s ability to do that is likely determined by its own structure and mechanical properties. Therefore, knowing exactly to which degree these properties are responsible for creating wear is crucial for being able to recognize different worked materials.

The results obtained in this study confirm previous results stating that it is possible to differentiate between the softest material (woods) and harder ones (referred as ABI: antler, bone, ivory) statistically. It is nonetheless important to note the statistical separation between the two types of wood, spruce (softer) and beech (harder). While wood is sometimes described as softer than antler ivory or bones, wood hardness depends on the wood species and the freshness of the wood. In our study the beech wood was closer in terms of hardness to ABI and not statistically different from them. Using basic surface topography, it does not seem possible to distinguish among antler, bone, ivory and beech wood. These observations confirm previously reported difficulties in visually differentiating surface modifications within this group, which are often reported together as ABI [66, 67].

An unresolved issue is that, even after 5 hours of work in our setup, harder materials created barely developed any surface modifications, while the wood samples abraded the stone surfaces almost completely smooth. Hence, for ivory, bone, and antler, it was impossible to distinguish the *before* and *after* use surfaces based on changes in *Sa*. In our previous work scraping beech wood [68], quantifiable differences in *Sa* were only documented with higher loads, of 90N and 100N. These loads were compatible with measurements we took during tasks conducted by humans in the lab [69]. However, here we were able to obtain changes in *Sa* for the same material, beech wood, even with the comparatively much lower load of 20N. The discrepancy can be attributed to the difference in the ability of that paper’s instrument (using focus variation) to measure surface modification and/or, given other teams’ successes using focus variation [11], to the lack of a standardized surface on which to measure it. As shown by Pfleging et al. [68], these new results confirm that load is not the factor contributing the most information to stone surface modification as it is possible to obtain an extended smoothing of the surface with a low load. However, it is also possible that with small loads much longer working durations are required to make noticeable changes to the stone surface. This could be due to other factors involved in the surface modifications, such as processes involving dislodging particles from the stone surface using friction heat. Natural lubricants in the worked material (e.g., grease in bone, water), the presence of abrasive particles such as the stone particles found in the residues, and mineral content of the worked material may also contribute to the modification of flint surfaces.

Finally, in addition to hardness, other factors, such as elasticity, fracture toughness, etc. can impact surface modification and polish formation and remain to be documented experimentally in future studies.

## Conclusion

As discussed in another paper related to this experimental setup [28], in this experiment polish is formed by the abrasion of the flint surface. Consequently, we expect that harder worked materials would be more effective at creating surface modifications. However, our experiment shows the softest worked materials produce the smoothest odified surfaces (i.e., the surface has the lowest arithmetical mean height (*Sa*)). Given these results, it is imperative that other mechanical properties of materials typically expected to occur in prehistoric tasks be studied. These could include the materials’ own surface roughness, elasticity, fracture toughness, the presence and brittleness of embedded grits, and others. In addition, longer work duration and/or higher loads (with different machines) can be tested to obtain more greater surface modifications on stone samples used on harder materials. Additionally, for this pilot study, materials were used in the dry condition, but saturation with water and added lubrication (e.g., fat) should be included in future studies.

## Supporting information

S1 Appendix Detailed results of the nano-indentation tests. (DOCX)

S1 File Detailed statistical analysis and code. (ZIP)
