# Supplementary material for: The effect of worked material hardness on stone tool wear
Source: PLoS One. 2022 Oct 20;17(10):e0276166. doi: 10.1371/journal.pone.0276166 (PMC9584531; doi:10.1371/journal.pone.0276166)
Supplement: S1 Appendix — (DOCX) [file pone.0276166.s001.docx]

­­

**S1 Appendix. Detailed results of the nano-indentation tests.**

Alice Rodriguez^1^*, Kaushik Yanamandra^2^, Lukasz Witek^3,4^, Zhong Wang^3^, Rakesh K. Behera^2^, Radu Iovita^1,5^*

^1^Anthrotopography Laboratory, Center for the Study of Human Origins, Department of Anthropology, New York University, New York, USA.

^2^Composite Materials and Mechanics Laboratory, Mechanical and Aerospace Engineering Department, New York University, Tandon School of Engineering, Brooklyn, New York, USA.

^3^Department of Biomaterials and Biomimetics, New York University College of Dentistry, New York, USA.
^4^Department of Biomedical Engineering, New York University Tandon School of Engineering, New York, USA.

^5^Department of Early Prehistory and Quaternary Ecology, Eberhard Karls University of Tübingen, Germany.

* Corresponding author

[aar596@nyu.edu](mailto:aar596@nyu.edu) (A.R.)

[iovita@nyu.edu](mailto:iovita@nyu.edu) (R. I.)

#
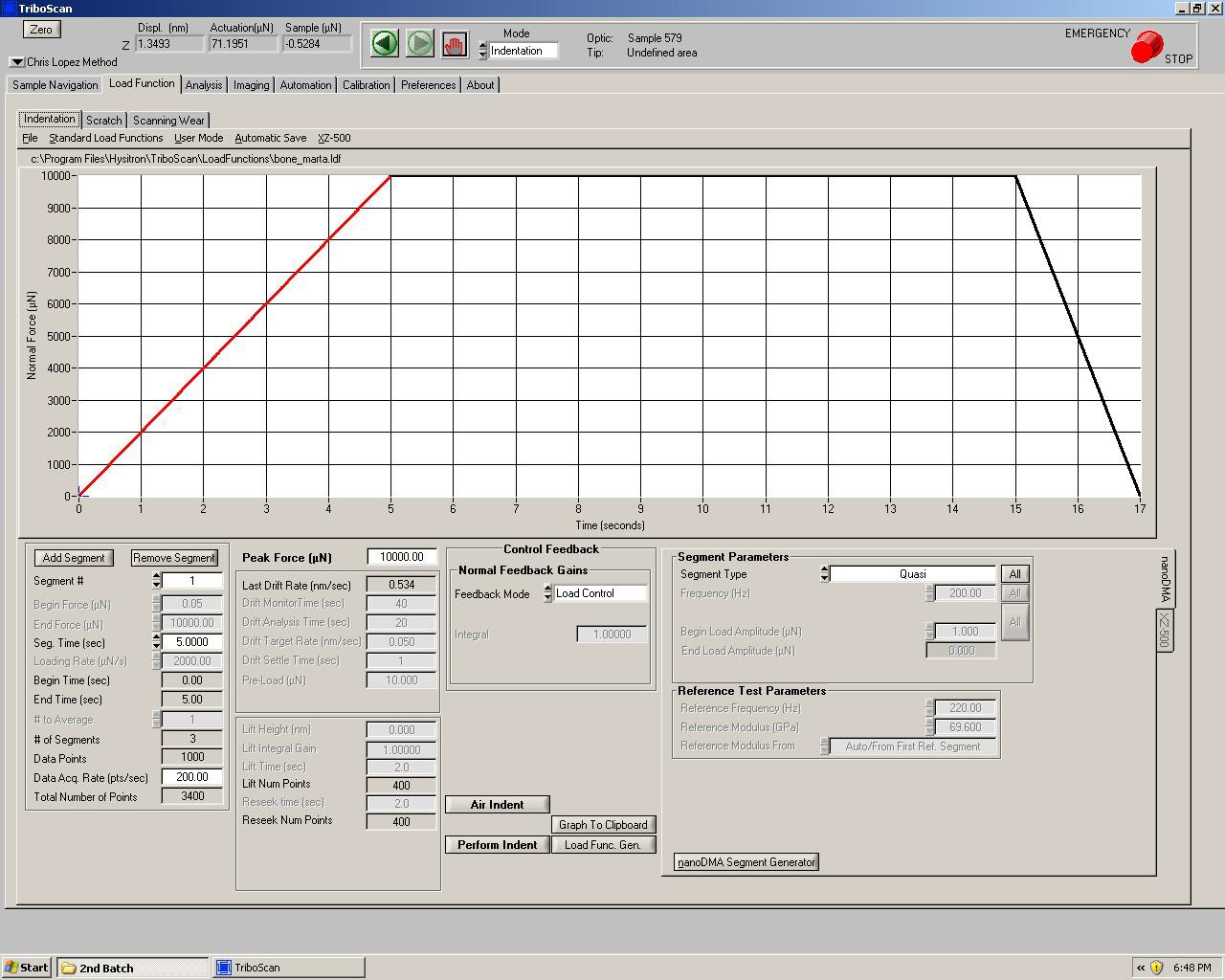
Loading Profile/Set Up

***y***

***x***

y= 4.0 um

x= 4.0 um

****Above Load Function used for ALL materials EXCEPT for Spruce-wood**

# Loading Function for Spruce-wood


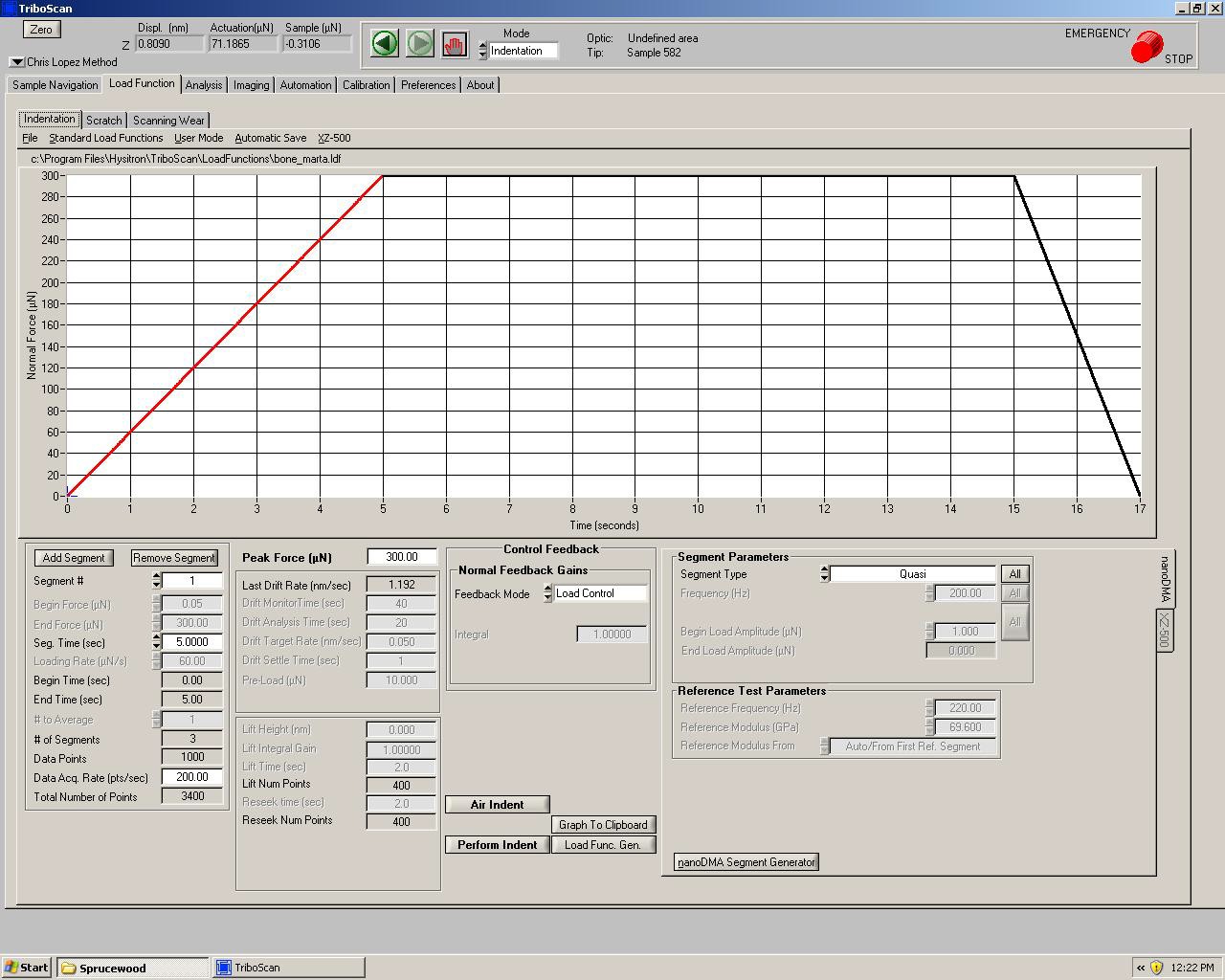


Flint samples


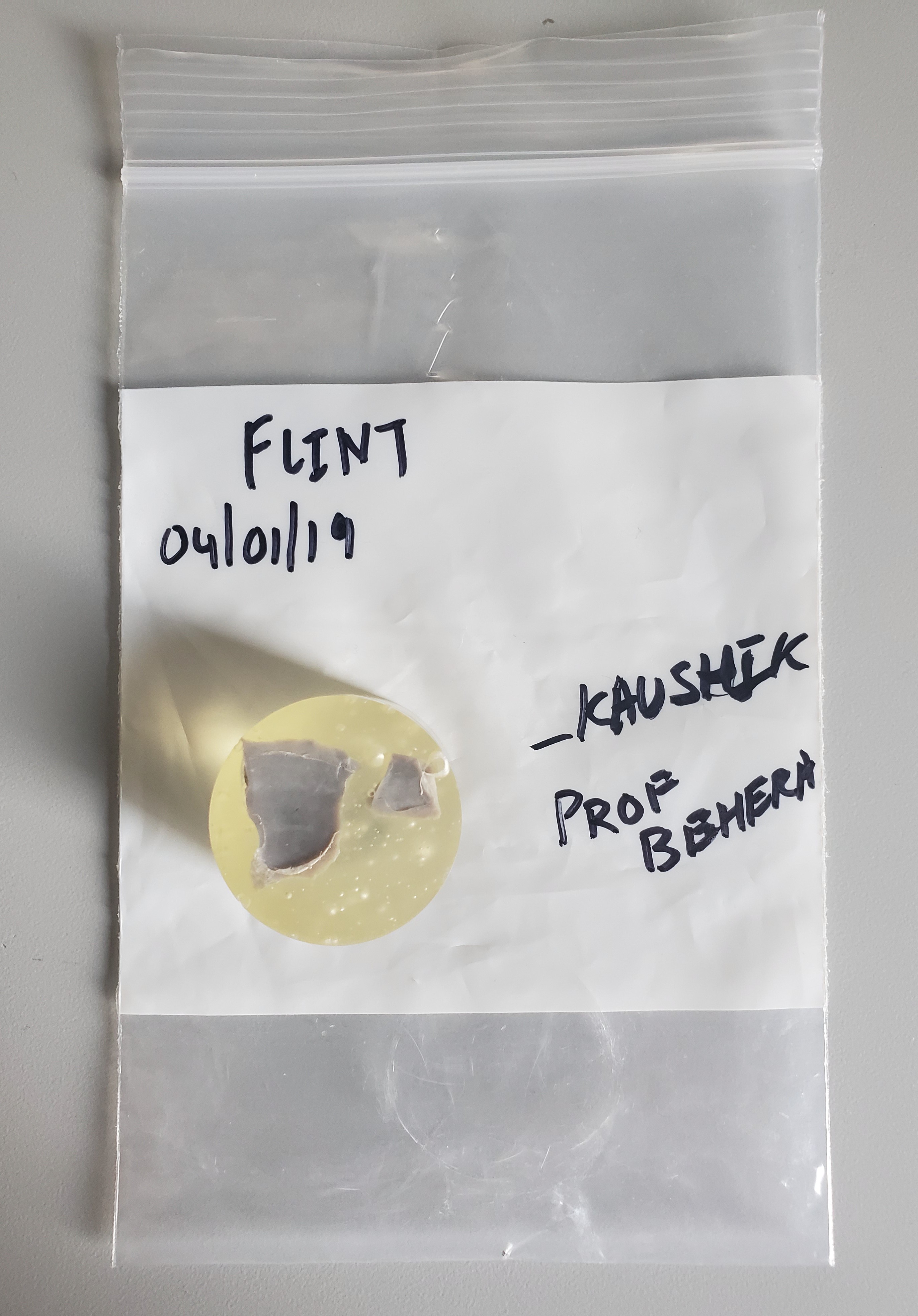
Smaller Flint


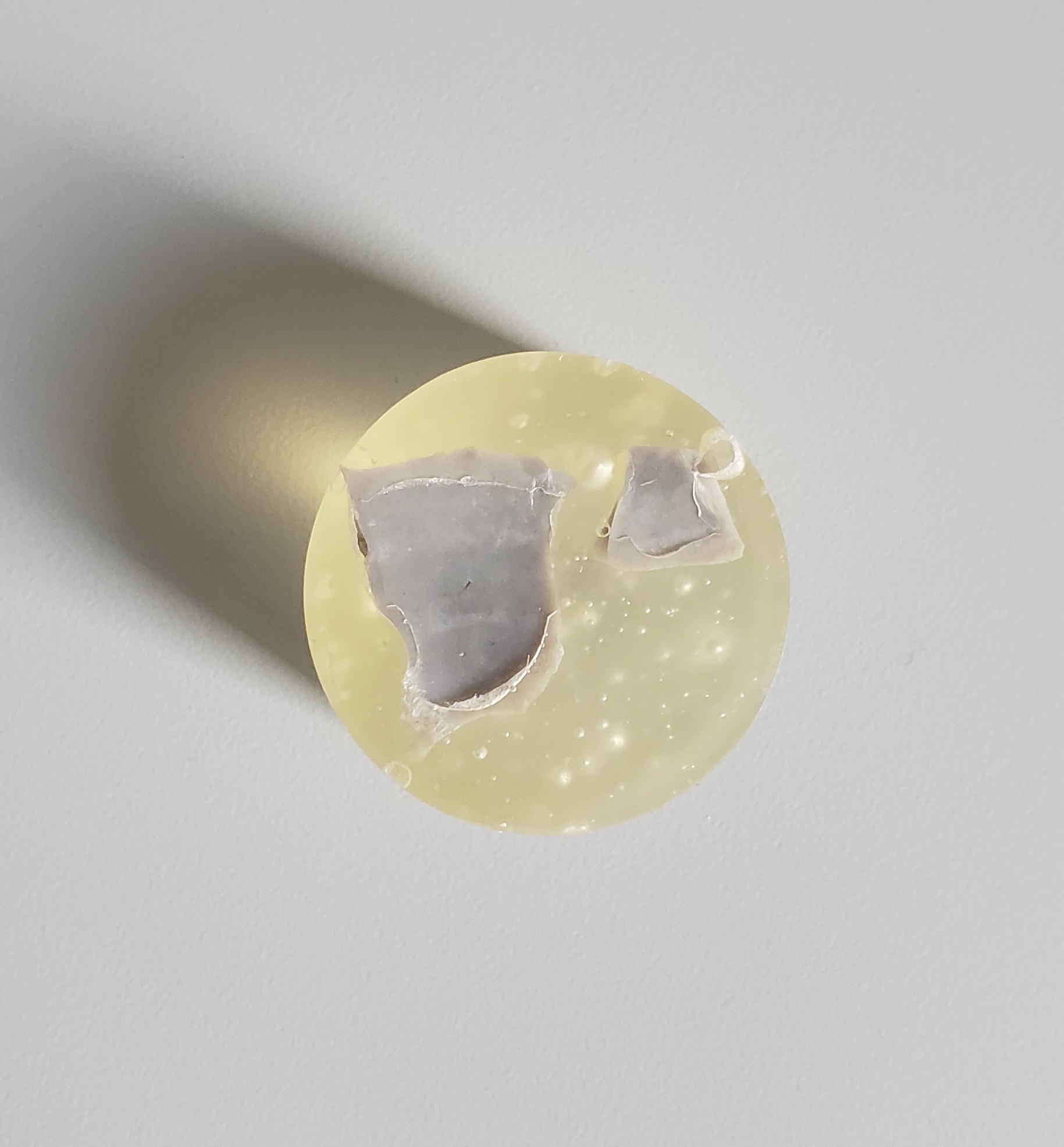


Larger Flint

Flint Indentation Curves

Flint Larger Area Flint Smaller Area


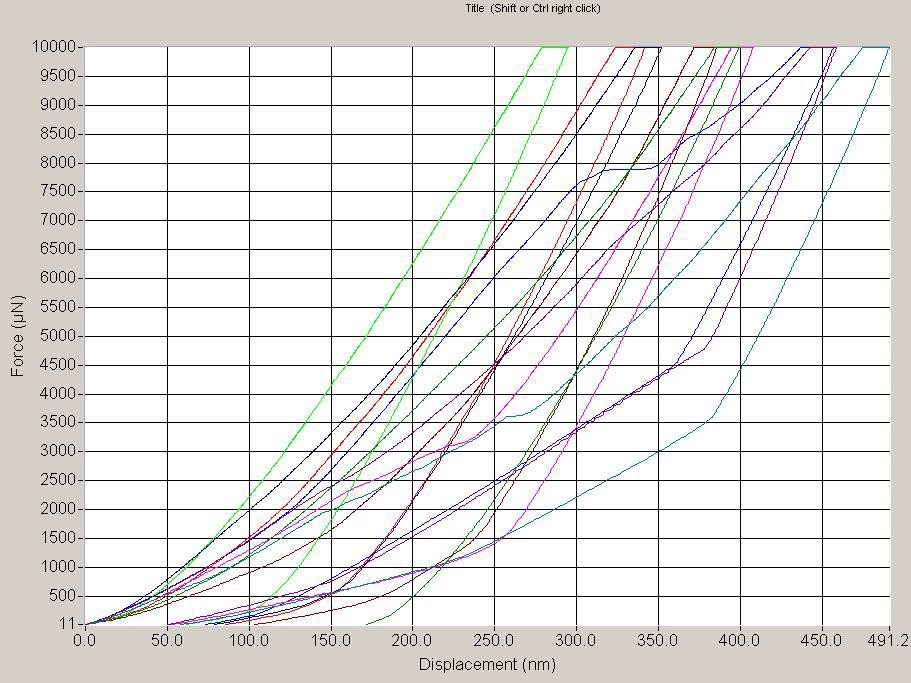

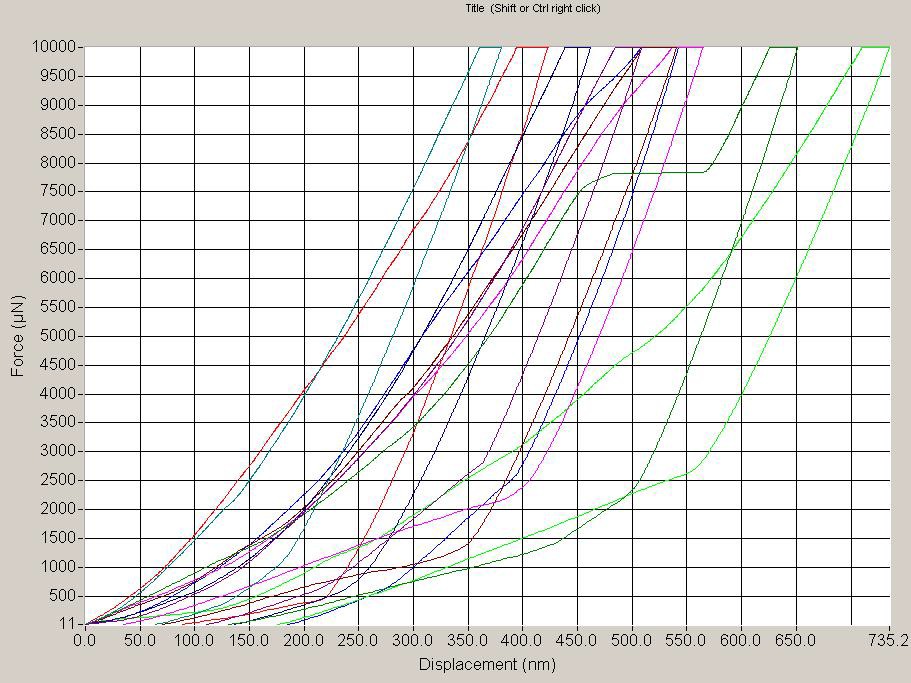


# Flint Er(GPa) & H(GPa)

Flint Larger Area Flint Smaller Area

| **Indent** | **Er(GPa)** | **H(GPa)** |
| --- | --- | --- |
| **1** | **45.558904** | **6.270127** |
| **2** | **31.606709** | **4.799645** |
| **3** | **51.660279** | **7.924633** |
| **4** | **43.075453** | **5.053764** |
| **5** | **47.195415** | **5.222133** |
| **6** | **41.43227** | **6.309974** |
| **7** | **40.402637** | **5.288314** |
| **8** | **39.871484** | **4.437459** |
| **9** | **30.099251** | **4.775906** |
| **Avg.** | **41.211** | **5.565** |
| **St. Dev.** | **6.943** | **1.093** |

| **Indent** | **Er(GPa)** | **H(GPa)** |
| --- | --- | --- |
| **1** | **37.948947** | **5.04903** |
| **2** | **38.536815** | **4.289196** |
| **3** | **31.949645** | **4.139238** |
| **4** | **37.37517** | **4.247003** |
| **5** | **31.762825** | **4.367318** |
| **6** | **34.323777** | **4.769343** |
| **7** | **37.766128** | **4.145938** |
| **8** | **36.857927** | **5.92682** |
| **9** | **38.554882** | **4.409819** |
| **Avg.** | **36.120** | **4.594** |
| **St. Dev.** | **2.728** | **0.583** |

# Ivory samples


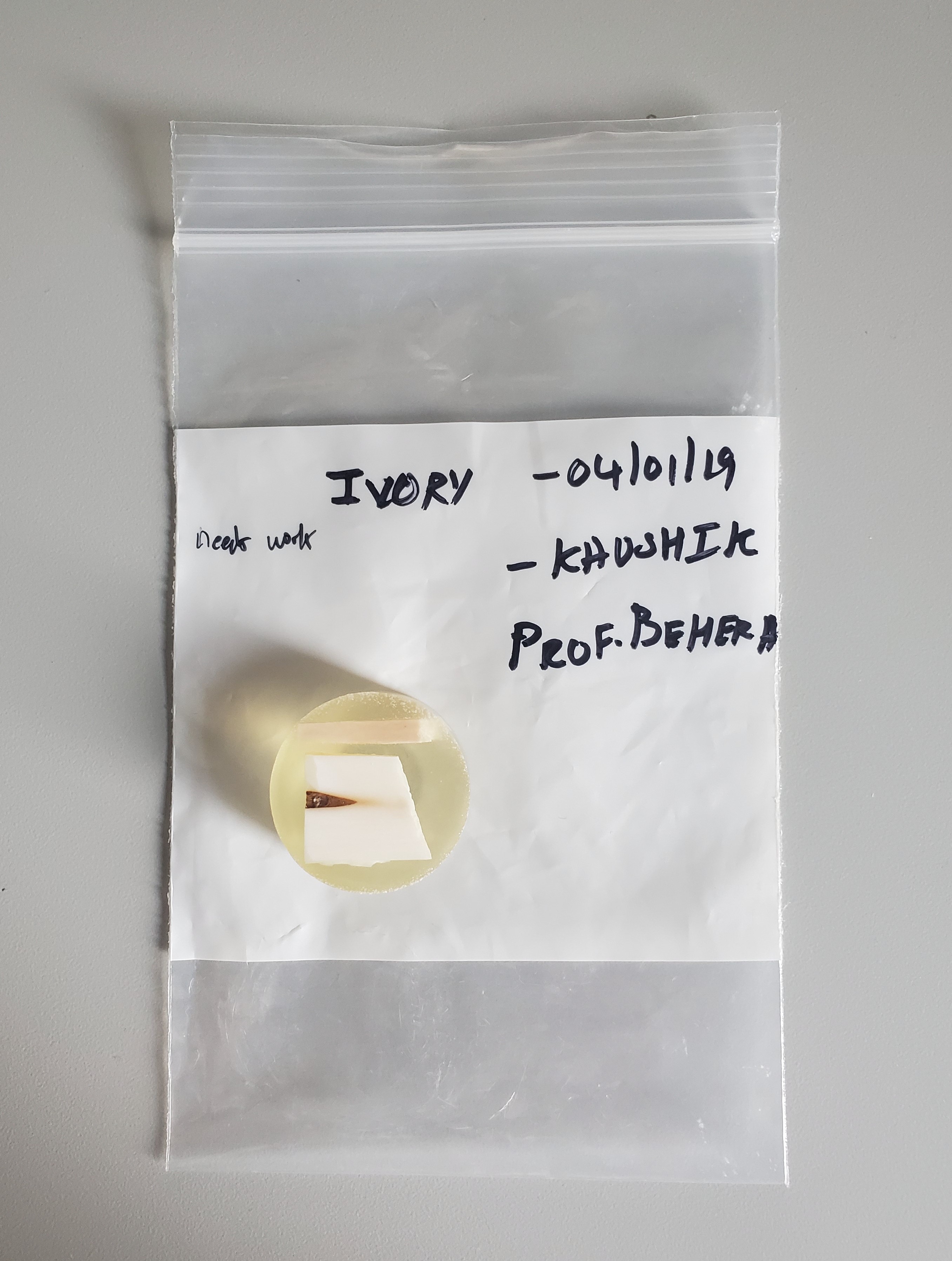
Larger Ivory Smaller Ivory


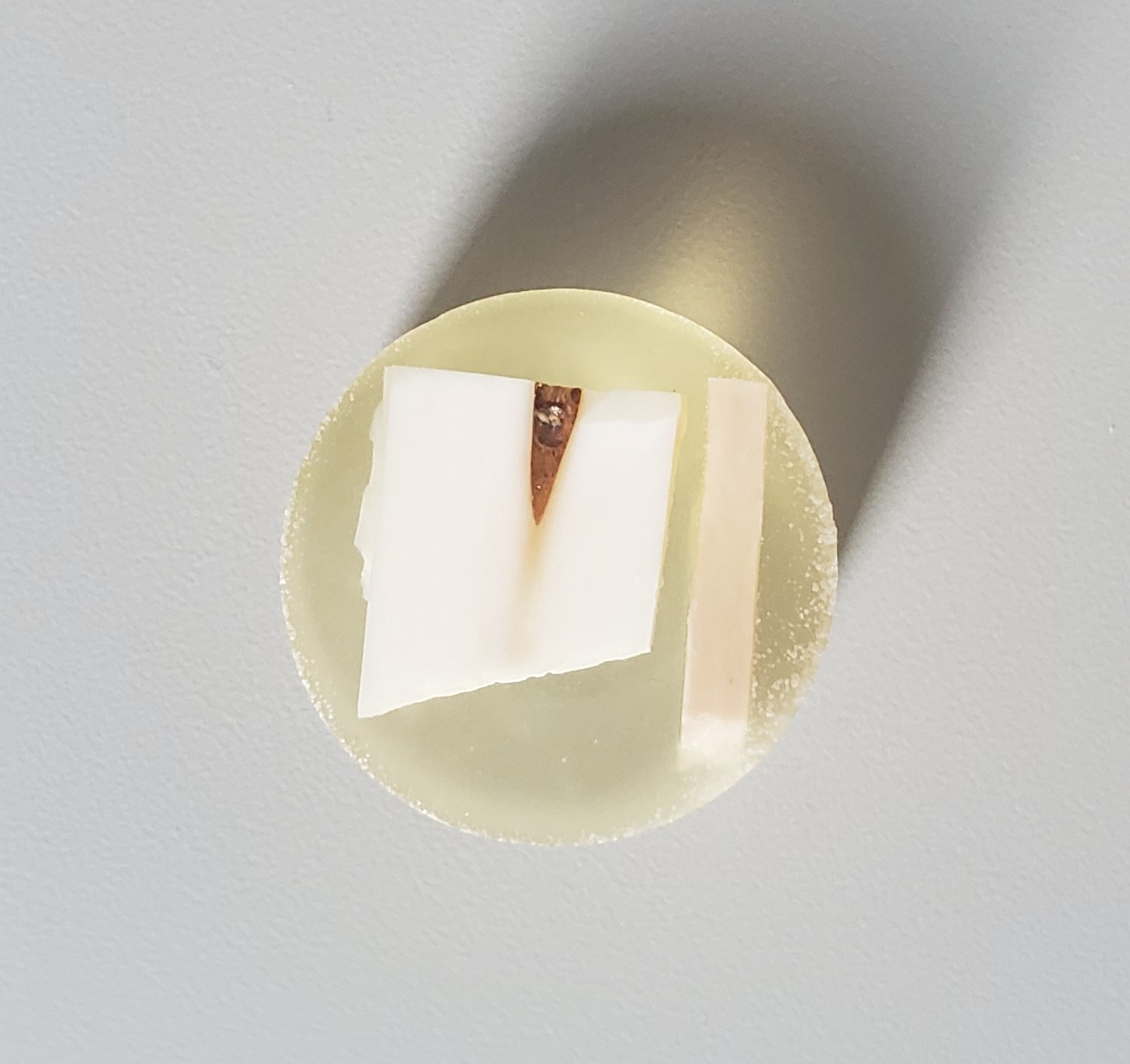


Ivory Indentation Curves

Ivory Larger Area Ivory Smaller Area


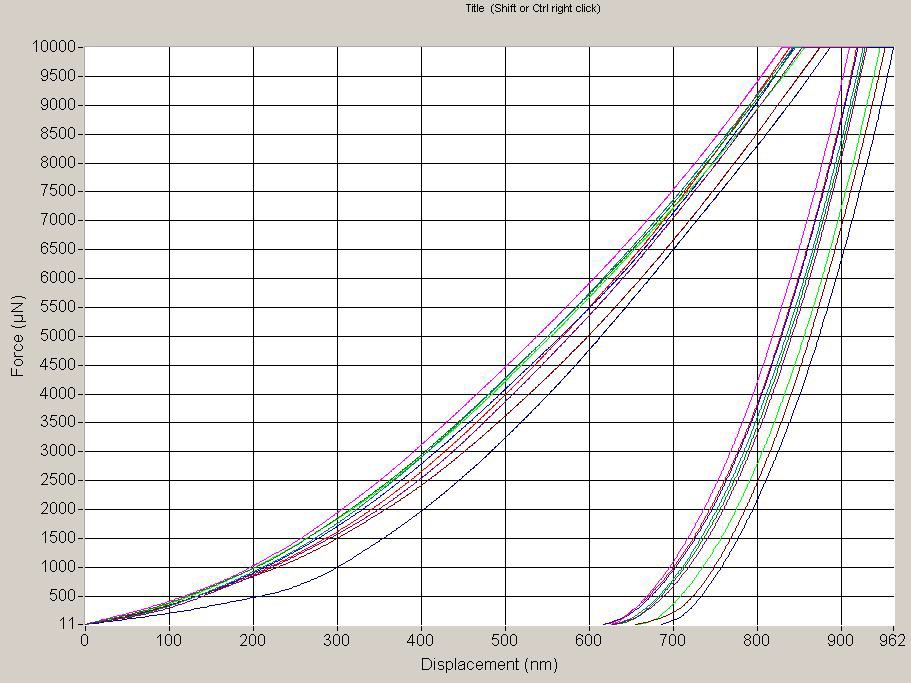

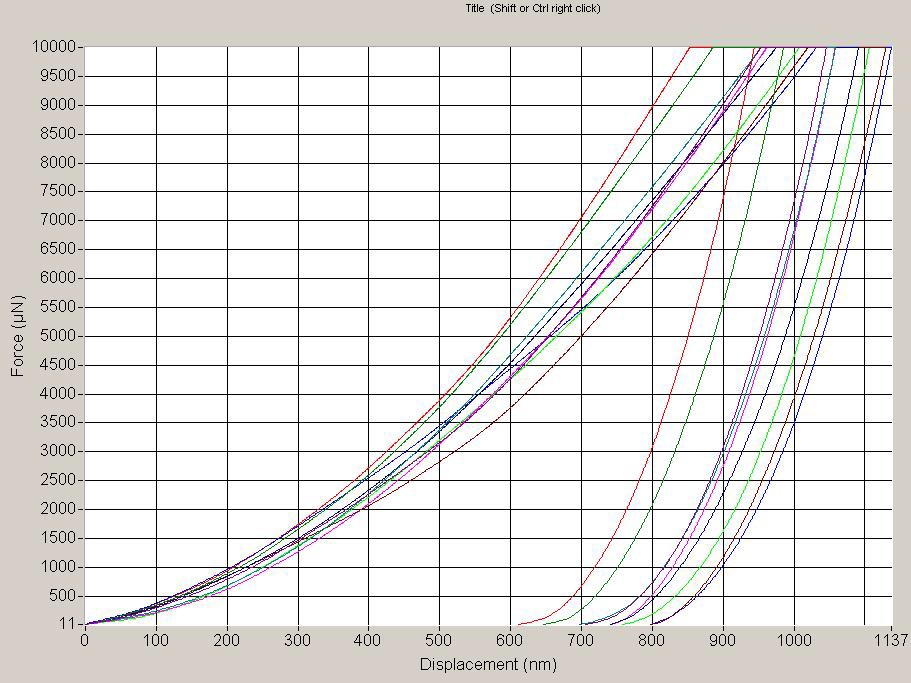


# Ivory Er(GPa) & H(GPa)

Larger Ivory Smaller Ivory

| **Indent** | **Er(GPa)** | **H(GPa)** |
| --- | --- | --- |
| **1** | **36.112338** | **4.097662** |
| **2** | **36.42405** | **4.095808** |
| **3** | **36.666962** | **4.059254** |
| **4** | **36.010383** | **4.107859** |
| **5** | **36.300293** | **4.051294** |
| **6** | **36.724923** | **4.030538** |
| **7** | **36.454701** | **4.085055** |
| **8** | **36.233454** | **4.089324** |
| **9** | **37.028375** | **4.079987** |
| **Avg.** | **36.439** | **4.077** |
| **St. Dev.** | **0.323** | **0.025** |

| **Indent** | **Er(GPa)** | **H(GPa)** |
| --- | --- | --- |
| **1** | **35.041842** | **4.06948** |
| **2** | **31.427003** | **3.521841** |
| **3** | **31.919692** | **3.649799** |
| **4** | **33.130843** | **3.812639** |
| **5** | **30.571815** | **3.579302** |
| **6** | **30.095188** | **3.736313** |
| **7** | **32.967296** | **4.010466** |
| **8** | **31.669926** | **3.829146** |
| **9** | **33.39925** | **3.847894** |
| **Avg.** | **32.247** | **3.784** |
| **St. Dev.** | **1.541** | **0.184** |

# Flint Type II samples


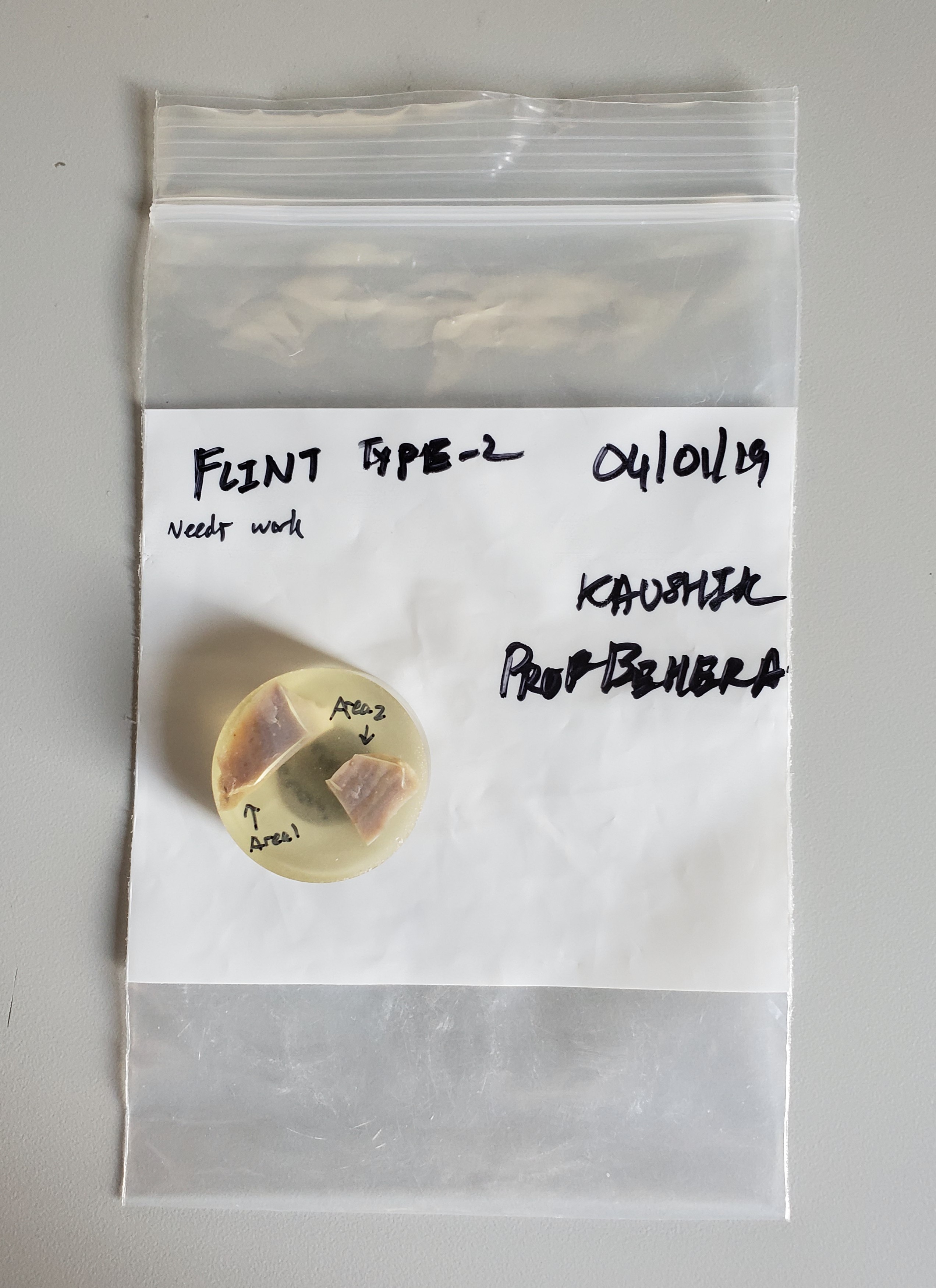

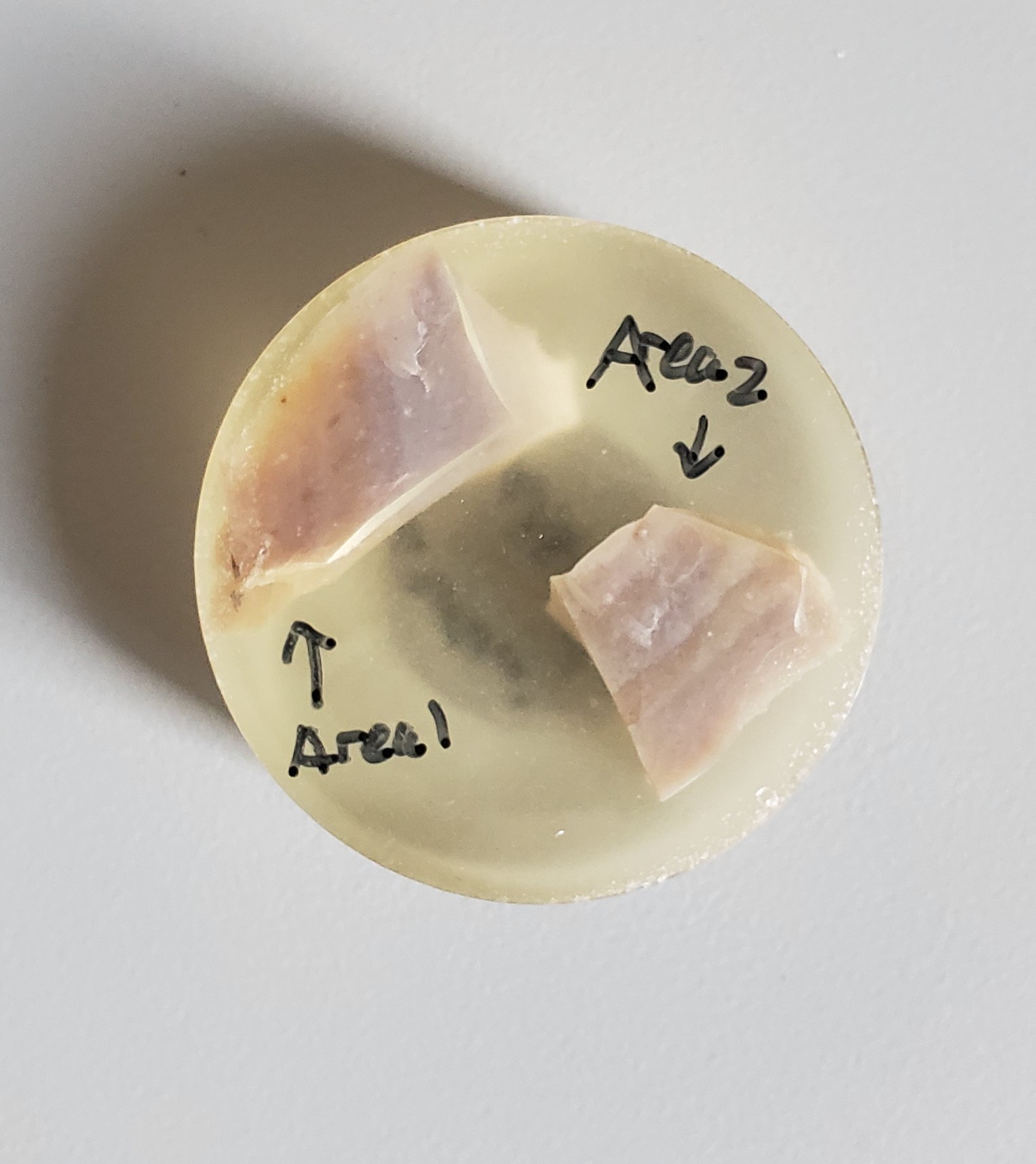


## Flint Type II Indentation Curve

Flint Type II Area 1 Flint Type II Area 2


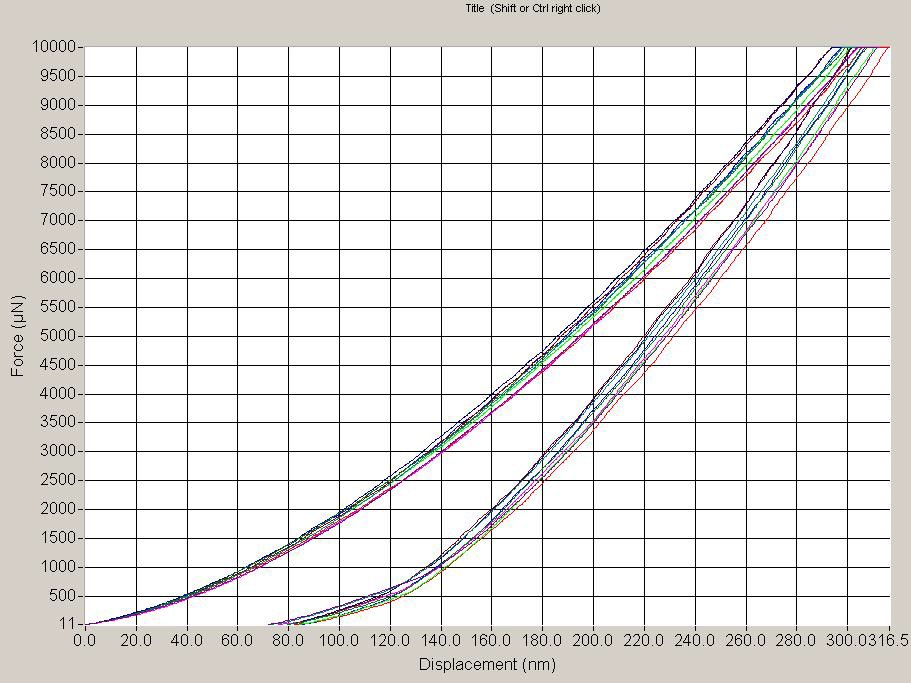

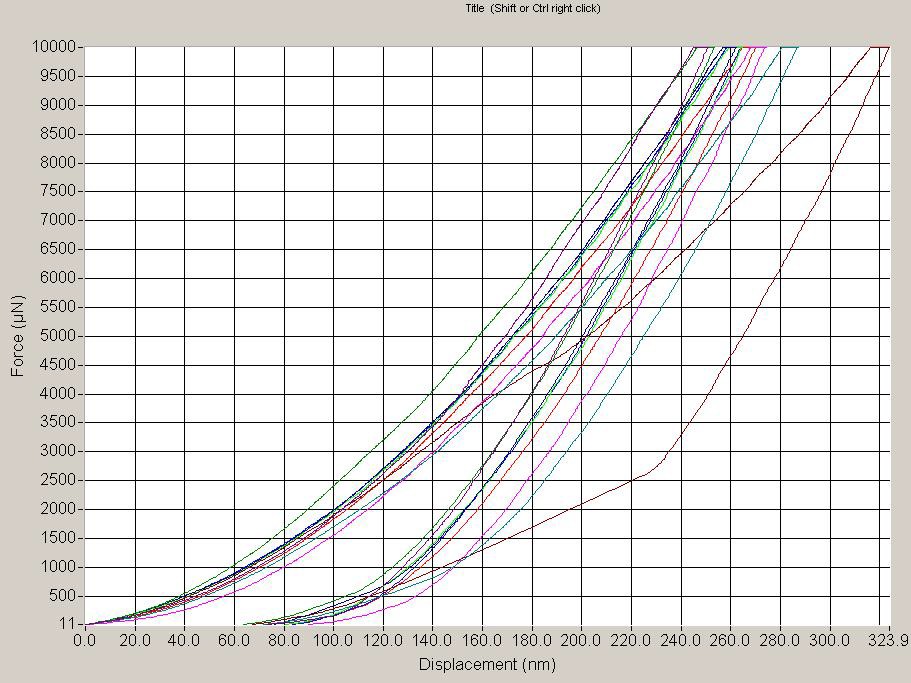


## Flint Type II Er(GPa) & H(GPa)

Flint Type II Area 1 Flint Type II Area 2

| **Indent** | **Er(GPa)** | **H(GPa)** |
| --- | --- | --- |
| **1** | **47.813** | **7.148194** |
| **2** | **49.805531** | **7.422021** |
| **3** | **50.239855** | **7.186294** |
| **4** | **48.406576** | **7.322133** |
| **5** | **50.687646** | **7.593115** |
| **6** | **51.629477** | **7.49724** |
| **7** | **50.616265** | **7.304802** |
| **8** | **49.416021** | **7.539853** |
| **9** | **49.182966** | **7.219727** |
| **Avg.** | **49.755** | **7.359** |
| **St. Dev.** | **1.193** | **0.161** |

| **Indent** | **Er(GPa)** | **H(GPa)** |
| --- | --- | --- |
| **1** | **69.378607** | **7.686407** |
| **2** | **70.223285** | **8.02563** |
| **3** | **71.055295** | **7.925008** |
| **4** | **73.215151** | **7.248752** |
| **5** | **70.982675** | **5.736898** |
| **6** | **70.837949** | **8.083244** |
| **7** | **76.663764** | **8.313469** |
| **8** | **67.303222** | **6.941884** |
| **9** | **76.835047** | **8.457254** |
| **Avg.** | **71.833** | **7.602** |
| **St. Dev.** | **3.196** | **0.851** |

# Deer Antler samples


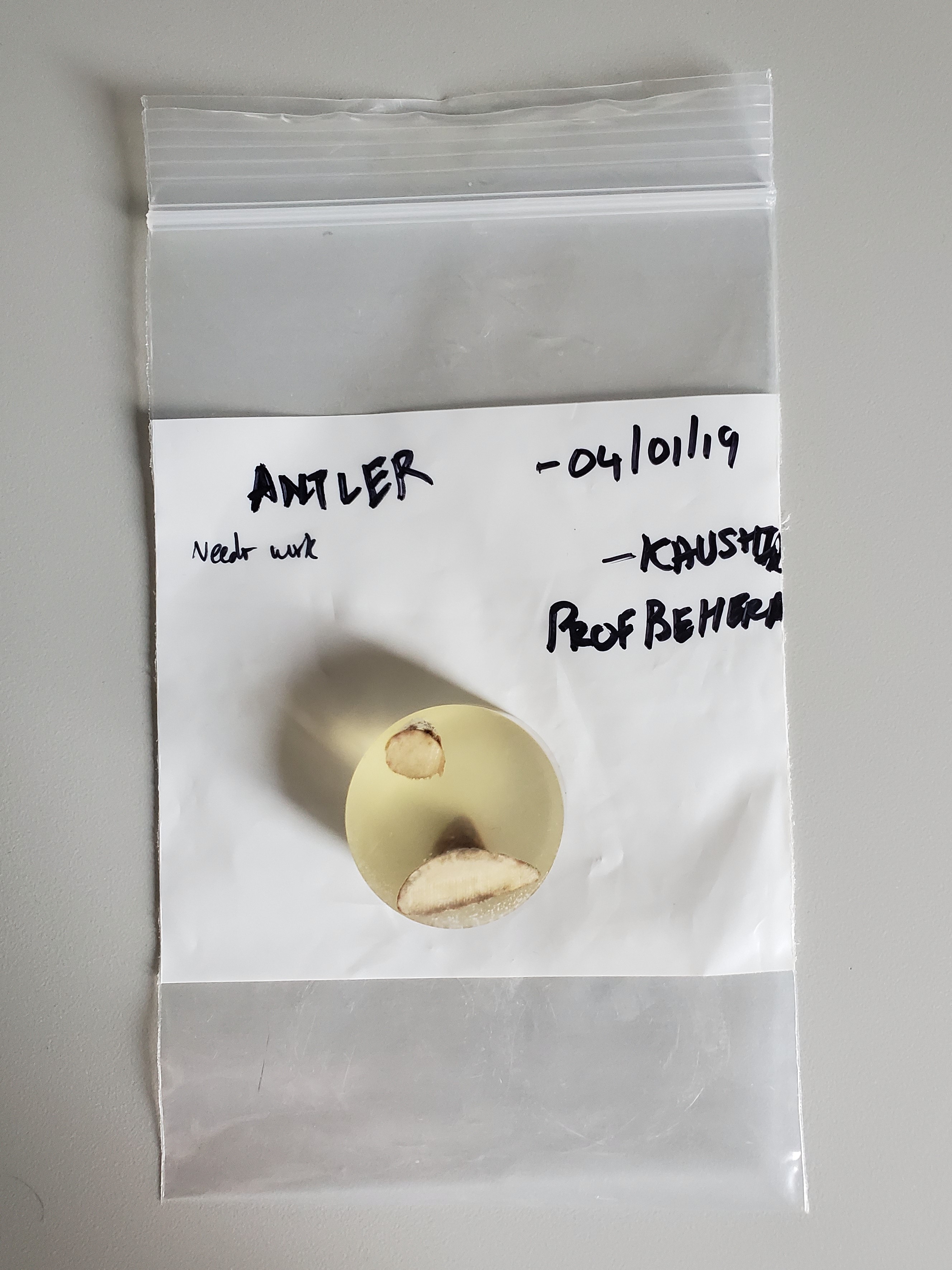
Area of Nano-Indent


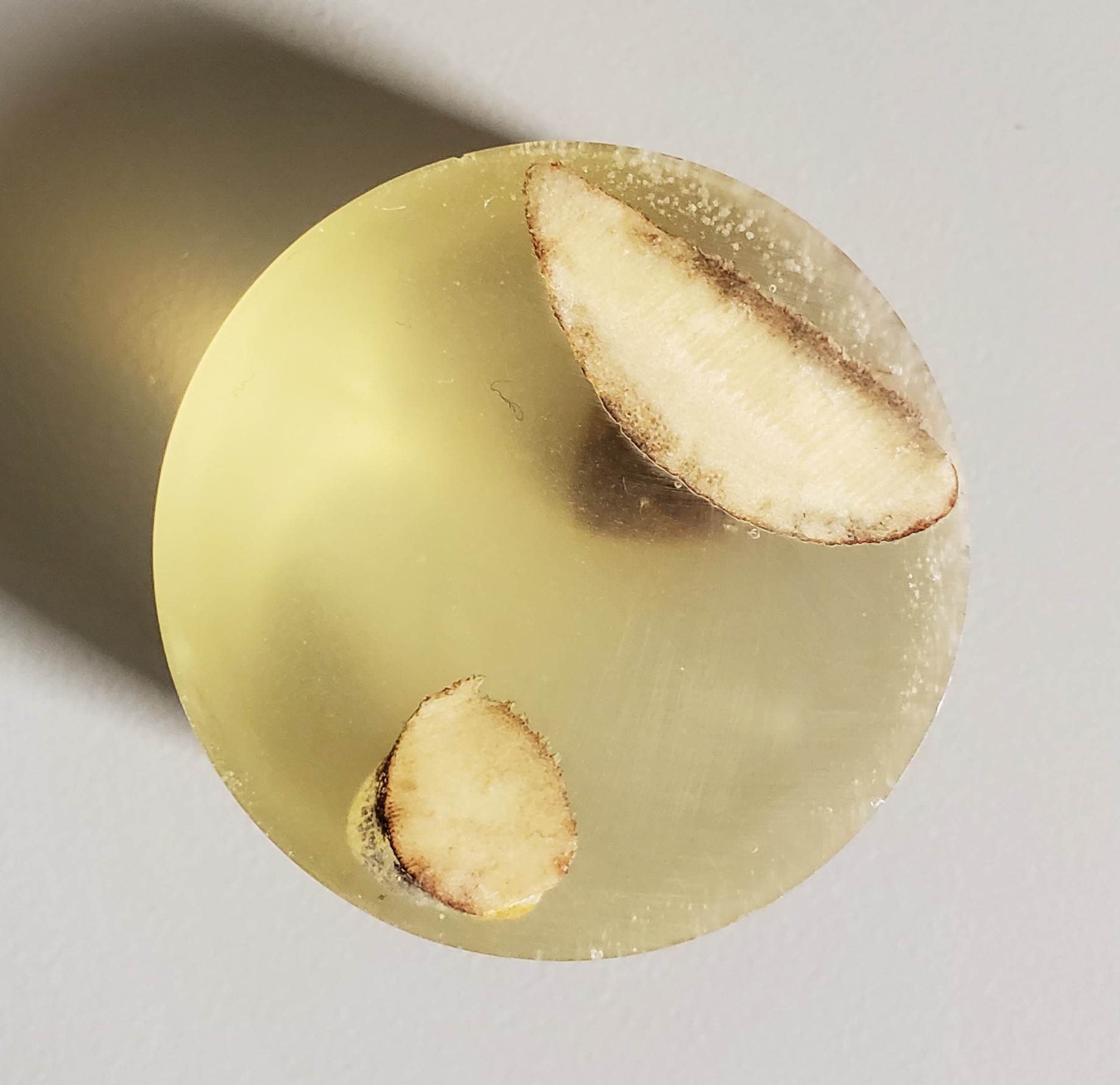


## Deer Antler Indentation Curve


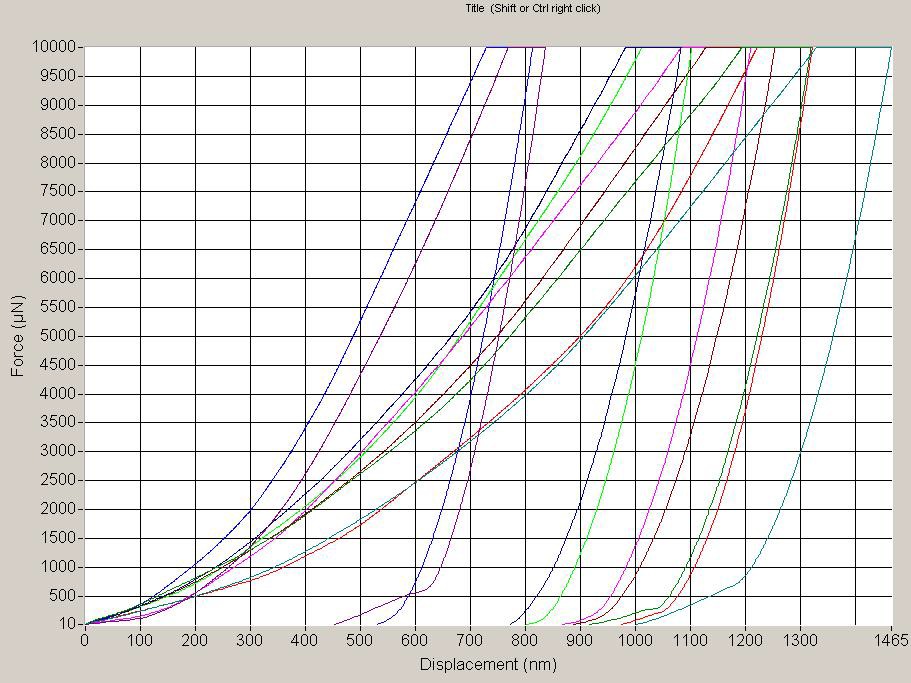


Deer Antler Er(GPa) & H(GPa)

Deer Antler

| **Indent** | **Er(GPa)** | **H(GPa)** |
| --- | --- | --- |
| **1** | **28.779763** | **2.623368** |
| **2** | **36.211751** | **4.150338** |
| **3** | **34.258747** | **3.633231** |
| **4** | **29.868289** | **3.203696** |
| **5** | **26.791464** | **3.043102** |
| **6** | **31.127443** | **3.754492** |
| **7** | **27.189043** | **2.675022** |
| **8** | **22.192257** | **2.050701** |
| **9** | **38.635946** | **4.146765** |
| **Avg.** | **30.562** | **3.253** |
| **St. Dev.** | **5.128** | **0.727** |

# Beechwood samples


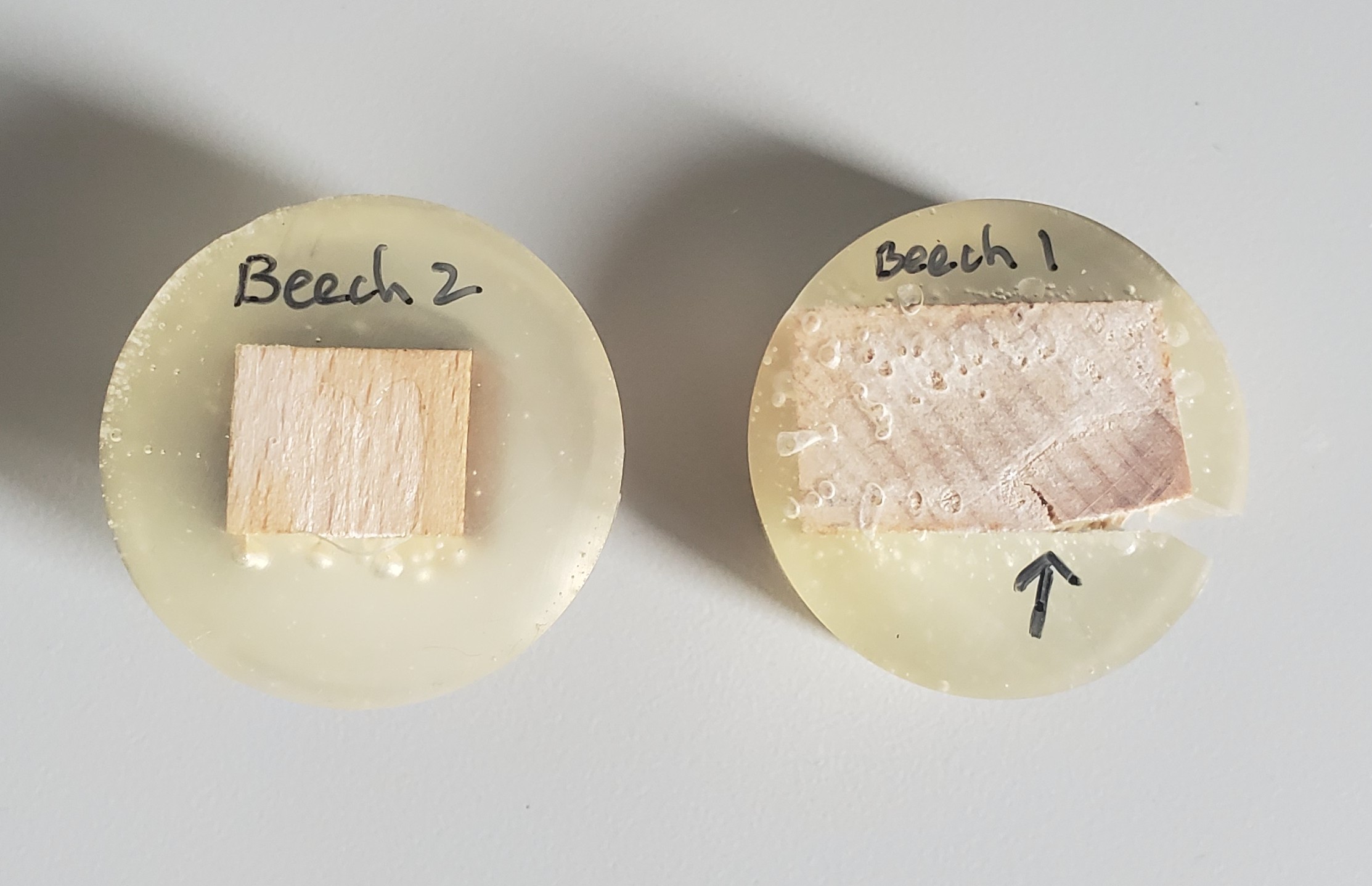


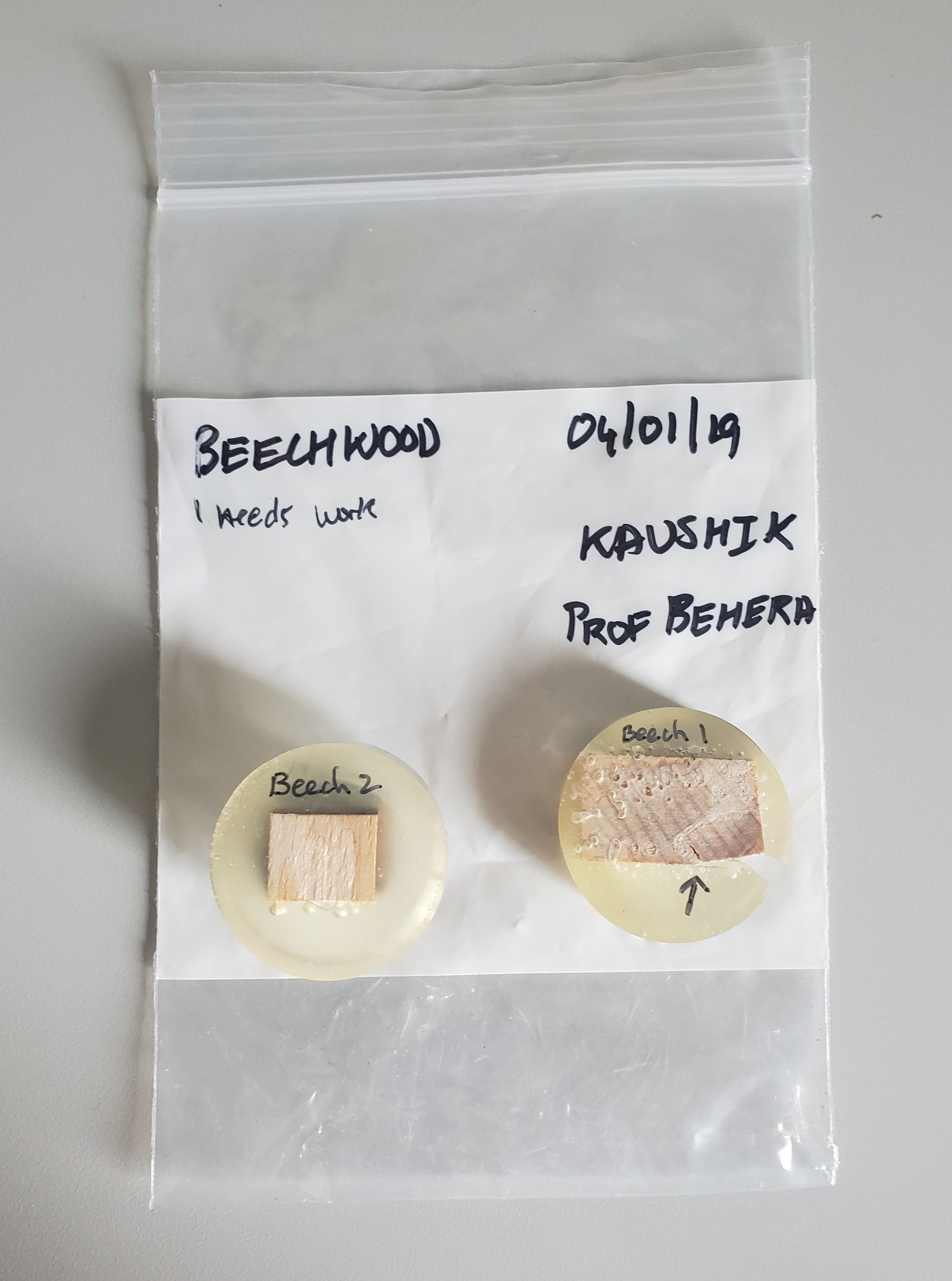
**Wood kept dry for proper hardness and modulus values**

## Beechwood Indentation Curve

Beechwood 1 Beechwood 2


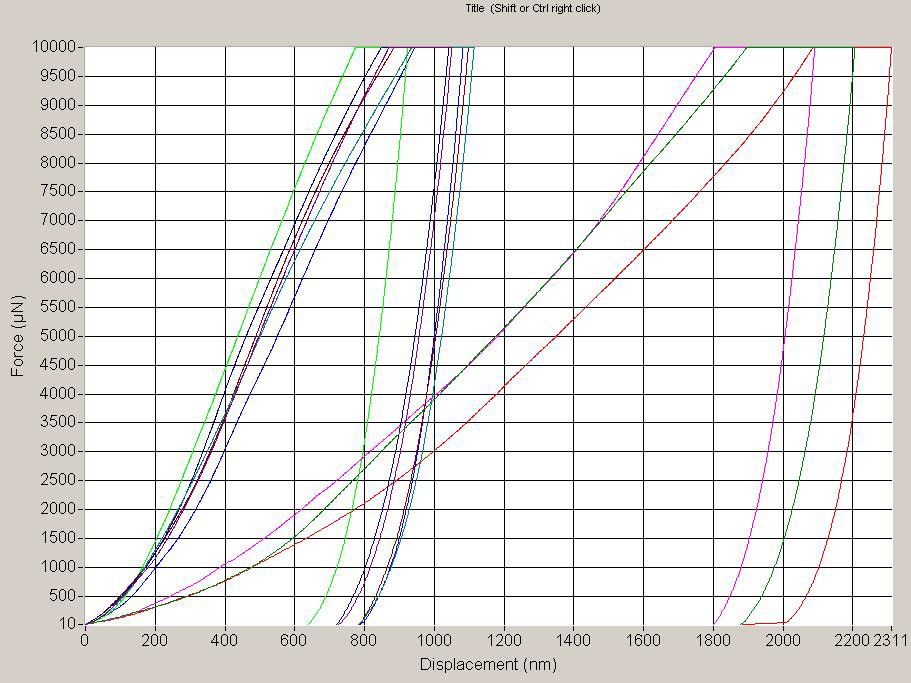

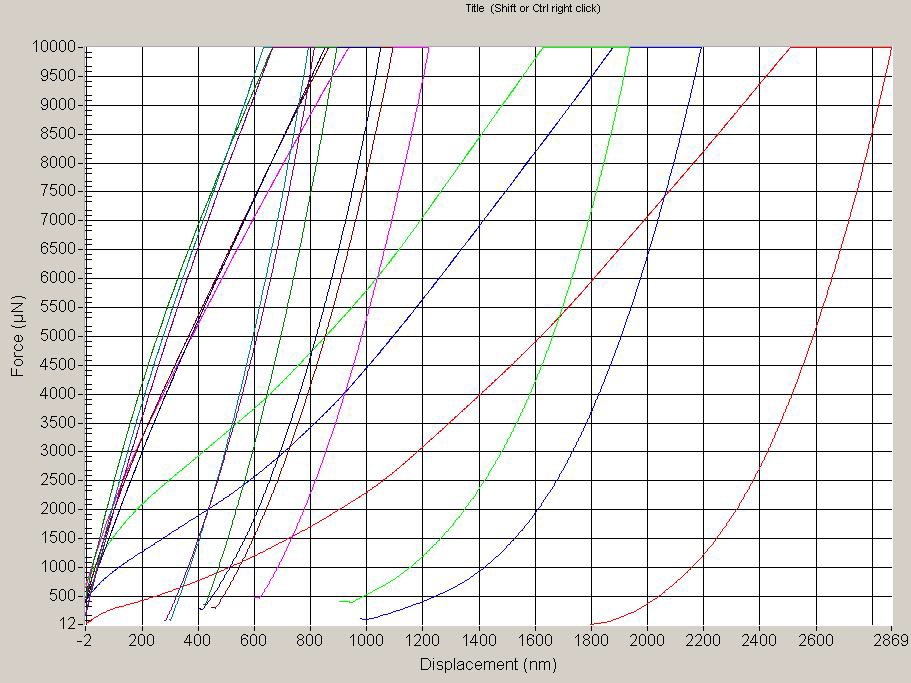


## Beechwood Er(GPa) & H(GPa)

Beechwood 1 Beechwood 2

| **Indent** | **Er(GPa)** | **H(GPa)** |
| --- | --- | --- |
| **1** | **13.408286** | **0.404912** |
| **2** | **37.895593** | **3.664547** |
| **3** | **40.081592** | **4.075533** |
| **4** | **14.475882** | **0.582239** |
| **5** | **32.997103** | **3.663294** |
| **6** | **33.03379** | **3.865389** |
| **7** | **12.70779** | **0.486182** |
| **8** | **32.781455** | **3.59708** |
| **9** | **33.678747** | **3.826944** |
| **Avg.** | **27.896** | **2.685** |
| **St. Dev.** | **11.062** | **1.652** |

| **Indent** | **Er(GPa)** | **H(GPa)** |
| --- | --- | --- |
| **1** | **2.980491** | **0.269551** |
| **2** | **4.920186** | **0.763626** |
| **3** | **6.316879** | **1.217147** |
| **4** | **13.096117** | **3.894199** |
| **5** | **13.566451** | **4.117693** |
| **6** | **14.04921** | **4.137729** |
| **7** | **15.798207** | **4.142441** |
| **8** | **16.164709** | **4.149262** |
| **9** | **15.80355** | **4.142613** |
| **Avg.** | **11.411** | **2.982** |
| **St. Dev.** | **5.181** | **1.692** |

# Spruce-wood samples


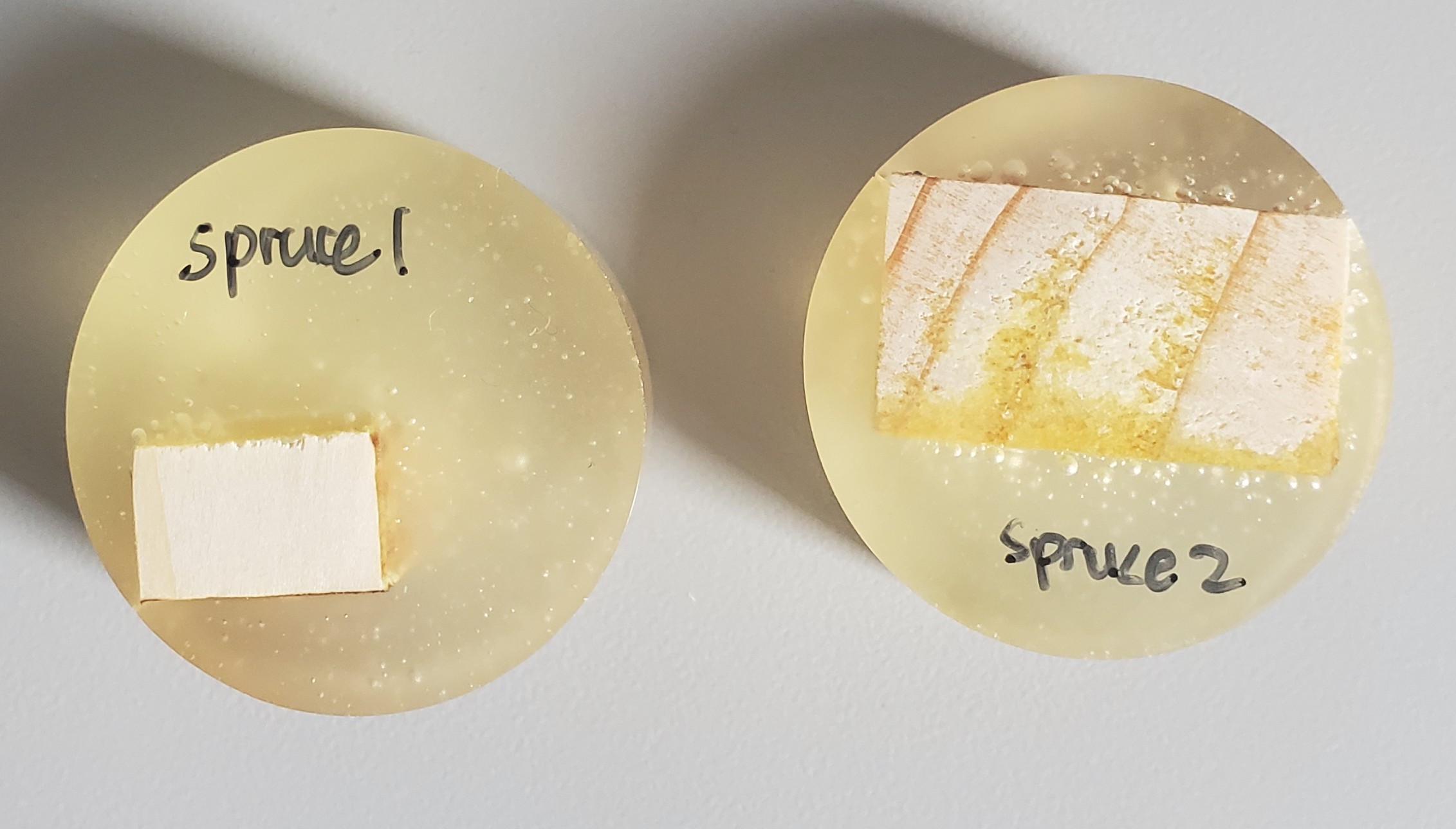


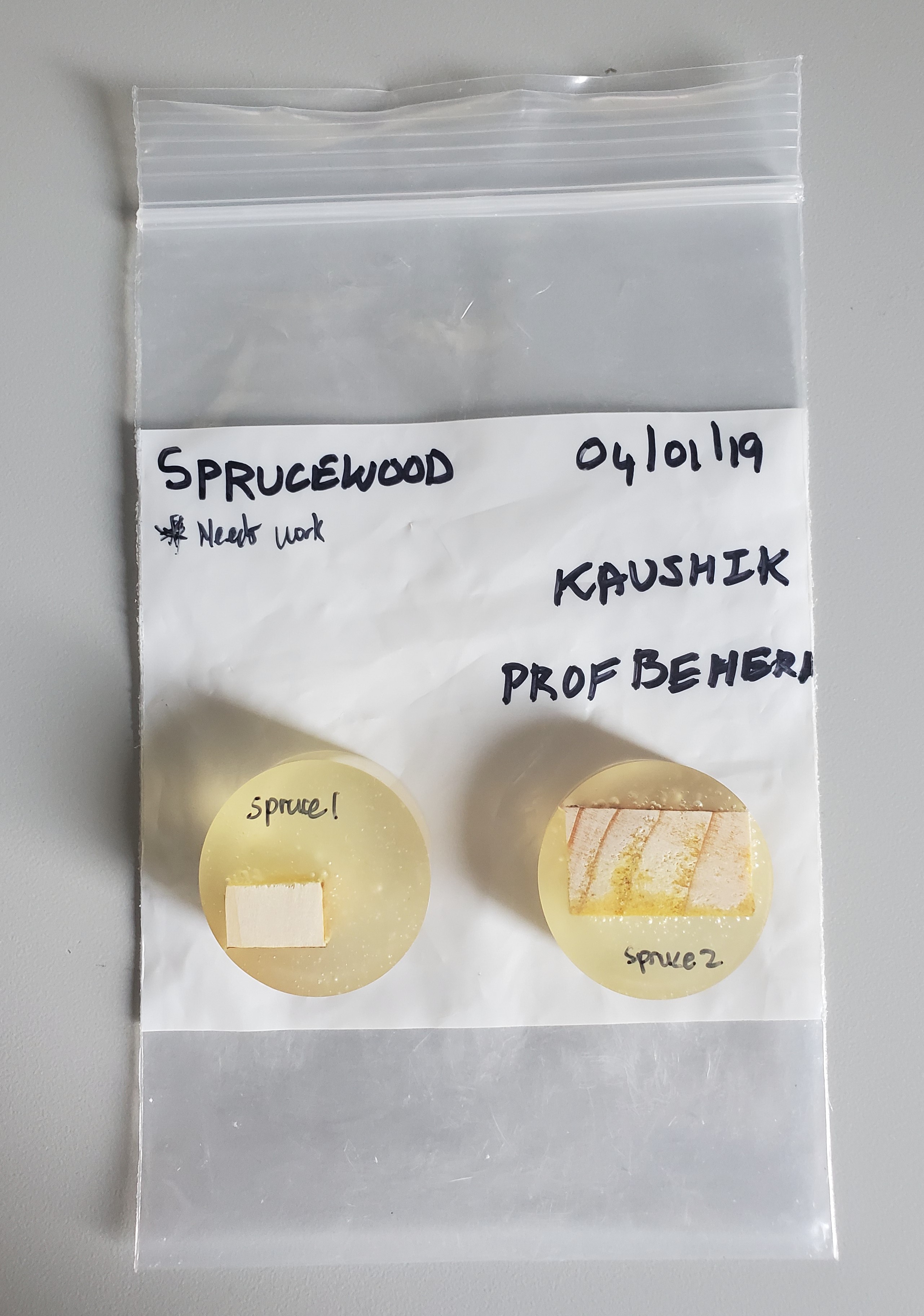
**Wood kept dry for proper hardness and modulus values**

## Spruce-wood Indentation Curve

Spruce-wood 1 Spruce-wood 2


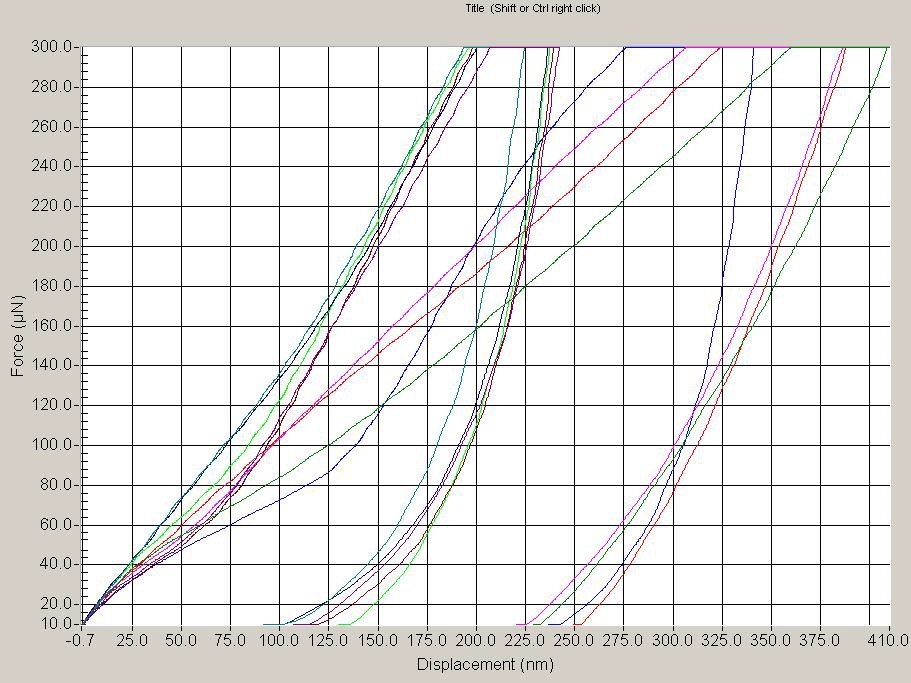

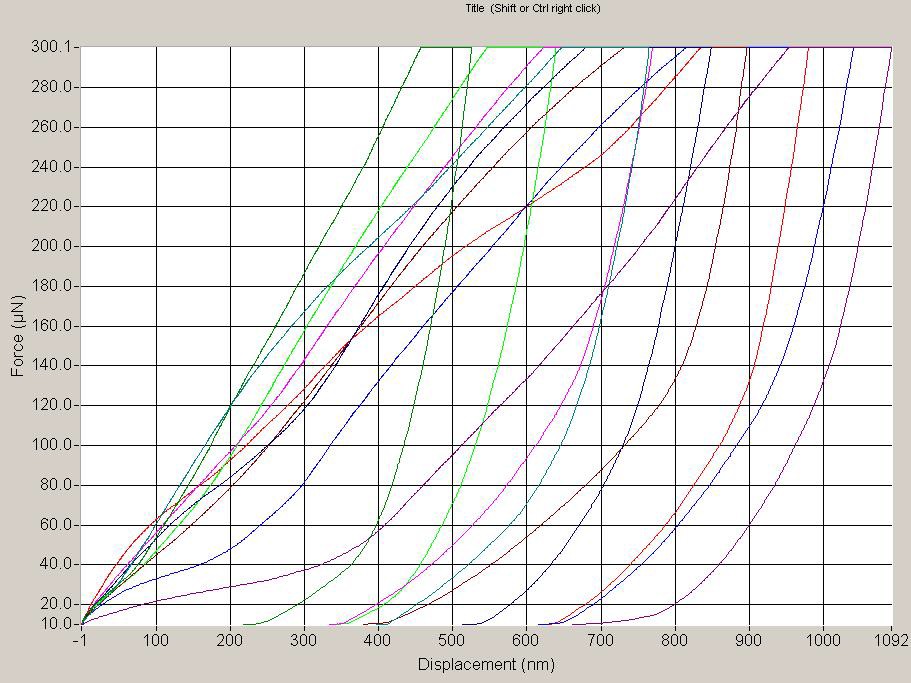


## Sprucewood Er(GPa) & H(GPa)

Spruce-wood 1 Spruce-wood 2

| **Indent** | **Er(GPa)** | **H(GPa)** |
| --- | --- | --- |
| **1** | **1.200649** | **0.118503** |
| **2** | **0.878406** | **0.116311** |
| **3** | **1.369703** | **0.12432** |
| **4** | **0.8513** | **0.124386** |
| **5** | **0.849243** | **0.123726** |
| **6** | **1.23408** | **0.124213** |
| **7** | **1.653575** | **0.127281** |
| **8** | **1.228907** | **0.124514** |
| **9** | **0.437492** | **0.114378** |
| **Avg.** | **1.078** | **0.122** |
| **St. Dev.** | **0.359** | **0.004** |

| **Indent** | **Er(GPa)** | **H(GPa)** |
| --- | --- | --- |
| **1** | **1.966068** | **0.146252** |
| **2** | **4.569977** | **0.147475** |
| **3** | **4.608175** | **0.20925** |
| **4** | **1.873717** | **0.147756** |
| **5** | **4.526345** | **0.207052** |
| **6** | **4.124629** | **0.213565** |
| **7** | **1.366272** | **0.149006** |
| **8** | **4.460774** | **0.227188** |
| **9** | **3.813141** | **0.210658** |
| **Avg.** | **3.479** | **0.184** |
| **St. Dev.** | **1.341** | **0.035** |

# Bone samples


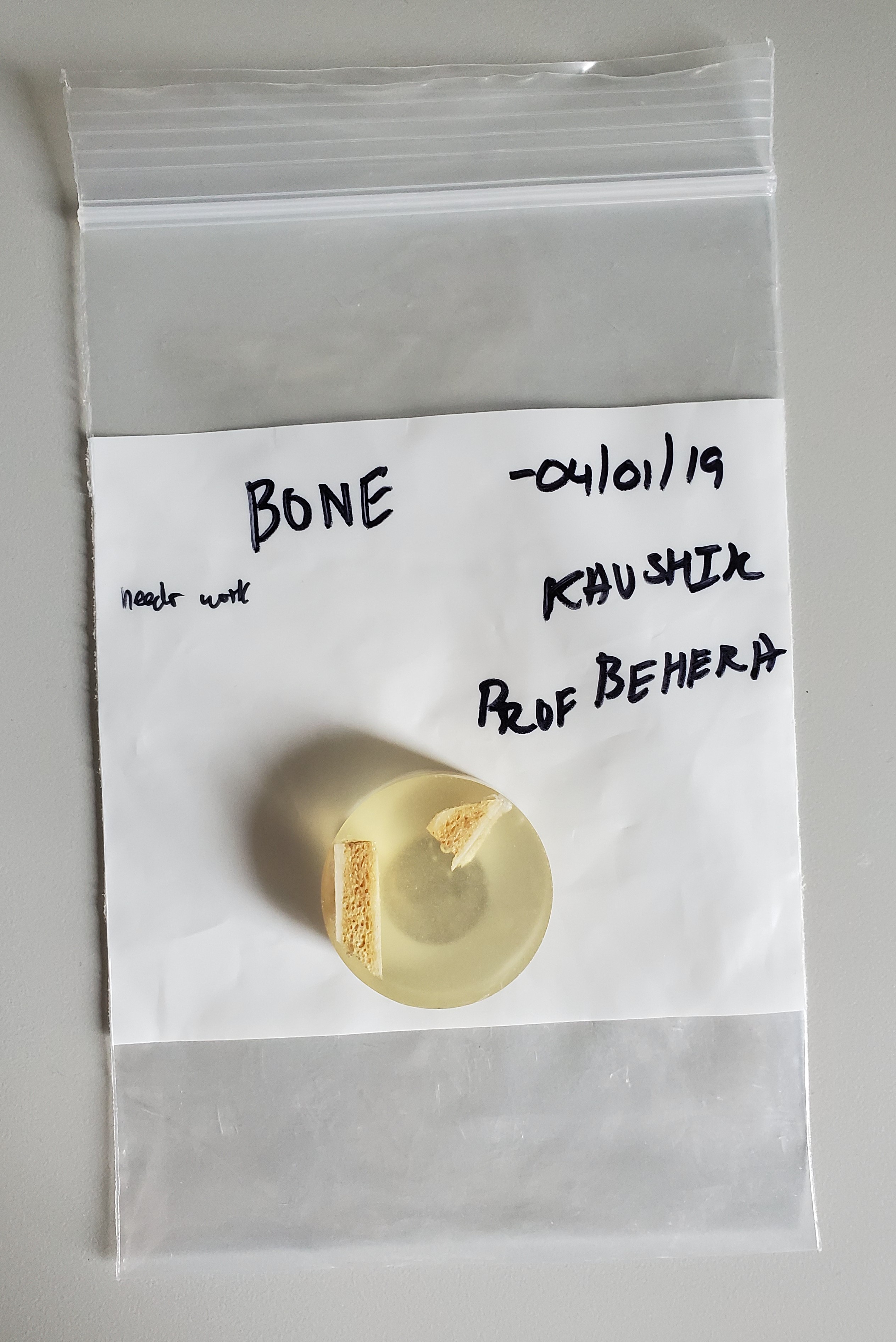
Bone Smaller Area


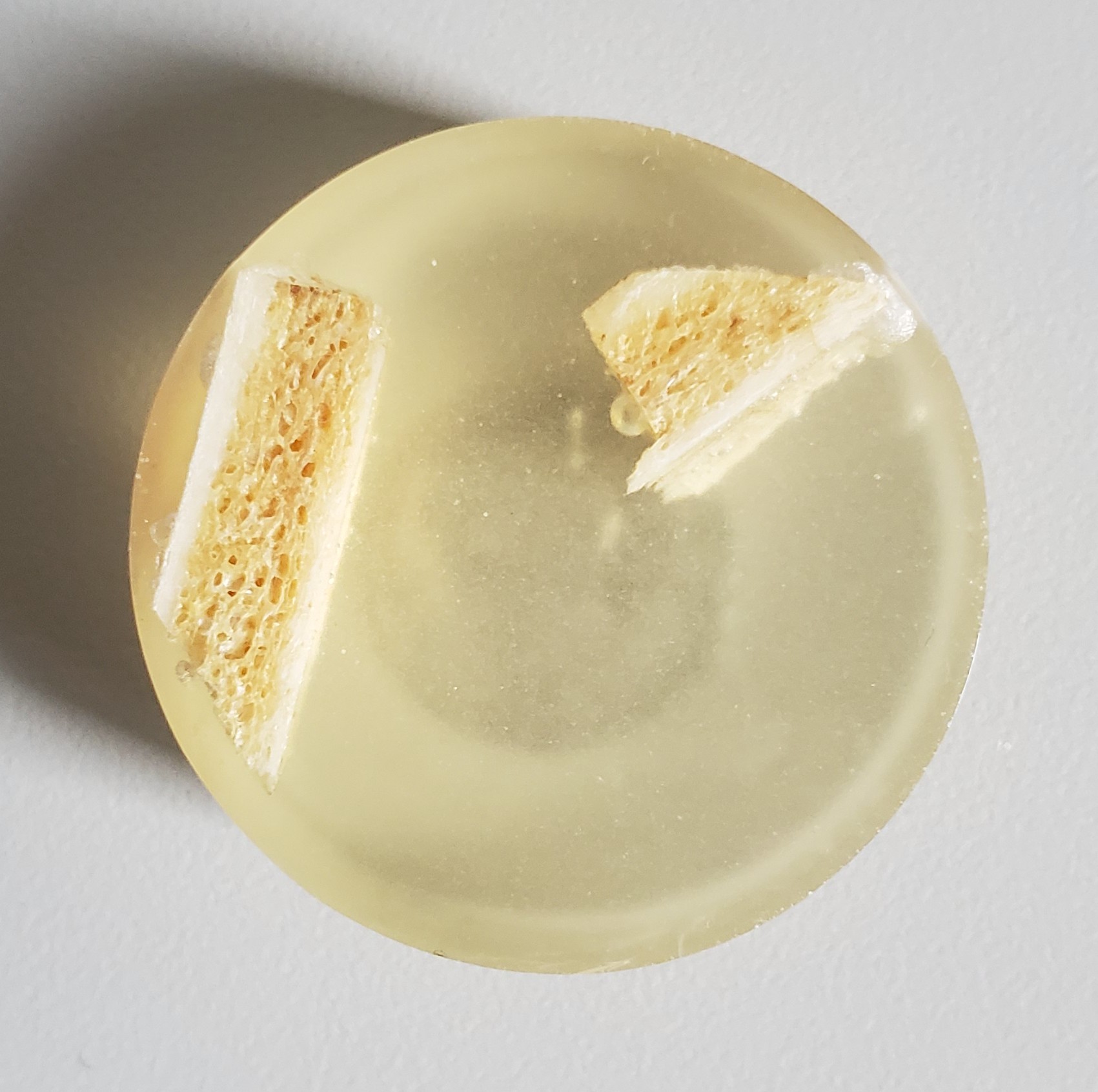


Bone Larger Area

Bone Indentation Curve

Bone Larger Area Bone Smaller Area


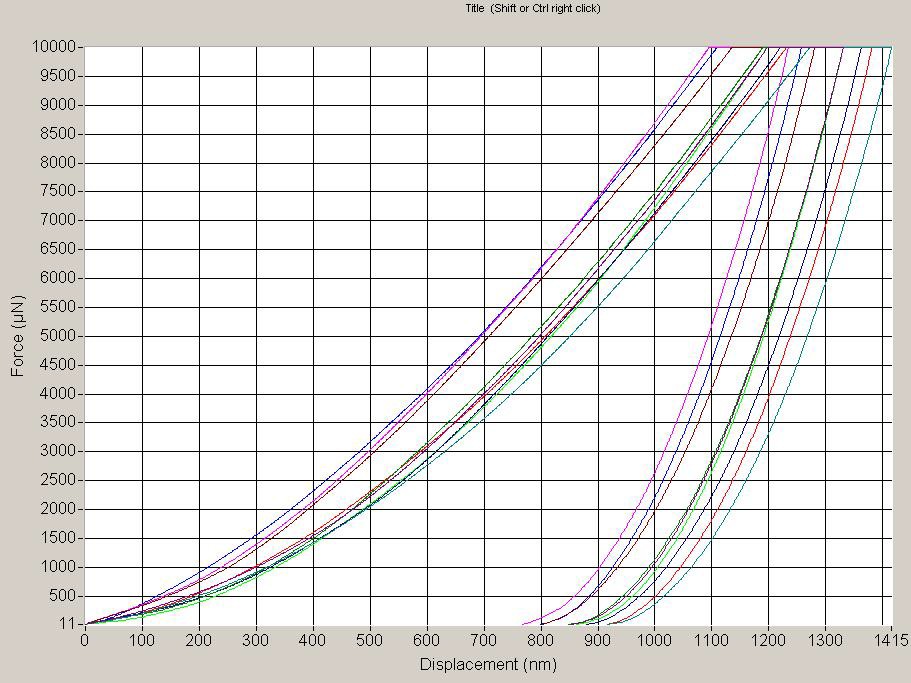

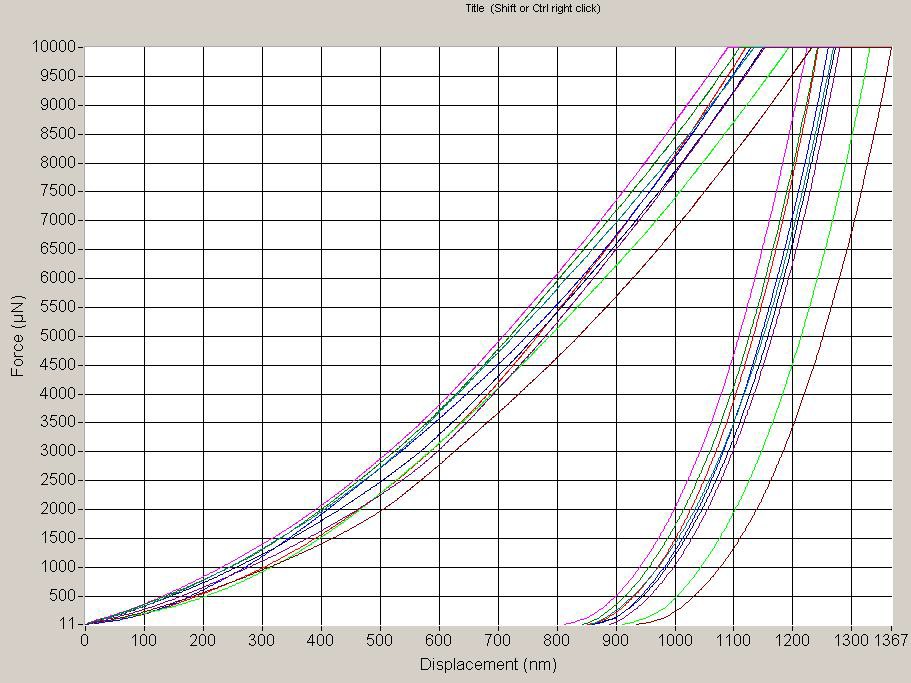


# Bone Er(GPa) & H(GPa)

Larger Bone Smaller Bone

| **Indent** | **Er(GPa)** | **H(GPa)** |
| --- | --- | --- |
| **1** | **18.365863** | **2.658053** |
| **2** | **20.48532** | **3.256486** |
| **3** | **19.692824** | **2.885814** |
| **4** | **20.99536** | **3.351673** |
| **5** | **19.569907** | **3.169682** |
| **6** | **18.457919** | **2.758778** |
| **7** | **19.079828** | **2.912595** |
| **8** | **17.286246** | **2.52419** |
| **9** | **19.3327** | **2.90449** |
| **Avg.** | **19.252** | **2.936** |
| **St. Dev.** | **1.128** | **0.277** |

| **Indent** | **Er(GPa)** | **H(GPa)** |
| --- | --- | --- |
| **1** | **25.8734** | **3.119402** |
| **2** | **24.789751** | **3.061351** |
| **3** | **22.824901** | **2.733714** |
| **4** | **25.452705** | **3.240315** |
| **5** | **22.079791** | **2.553501** |
| **6** | **24.7219** | **3.002075** |
| **7** | **24.927498** | **3.159103** |
| **8** | **23.928095** | **3.04381** |
| **9** | **24.378896** | **2.966439** |
| **Avg.** | **24.331** | **2.987** |
| **St. Dev.** | **1.217** | **0.216** |
