# Supplementary material for: The effect of worked material hardness on stone tool wear
Source: PLoS One. 2022 Oct 20;17(10):e0276166. doi: 10.1371/journal.pone.0276166 (PMC9584531; doi:10.1371/journal.pone.0276166)
Supplement: S1 File — (ZIP) [file pone.0276166.s002.zip › Markdown Version/triboanalysisV6.html]

 

 

 

 
 
 


 

 

 triboanalysis 

 
 
 
 
 
 
 
 
 
 
 

 

 
 
 


 


 


 

 

 


 

 


 


 


 triboanalysis 
 Alice Rodriguez 
 October 19, 2020 

 


 
 Abstract 
 The identification of ancient worked materials is one of the fundamental goals of lithic use wear analysis and one of the most important parts of understanding how stone tools were used in the past. Given the documented overlaps in wear patterns generated by different materials, it is imperative to understand how individual materials' mechanical properties might influence wear formation. Because isolating physical parameters and measuring their change is necessary for such an endeavor, controlled (rather than replicative) experiments combined with objective measurements of surface topography are necessary to better grasp how polish formed on stone tools. Therefore, we used a tribometer to wear natural flint surfaces against five materials (bone, antler, beech wood, spruce wood, and ivory) under the same force, and speed, over one, three, and five hours. The study aimed to test if there is a correlation between polish formation and the hardness of the worked material. We measured each raw material's hardness using nano-indentation test, and we compared the surface texture of the polished flint bits using a 3D optical profilometer. The interfacial detritus powder was analyzed with a scanning electron microscope to look for abraded flint particles. We demonstrate that, contrary to expectation, softer materials, such as wood, create smoother polishes than hard ones, such as ivory. 
 
 
 Introduction 
 Along with the study of ancient residues, microscopic use wear analysis (MWA) is one of the two major and complementary methods of lithic traceology [1], the science of forensically interpreting ancient stone tool use. Despite its immense interpretive potential, its early spectacular results [e.g., 2] were tempered by the blind testing crisis of the 1980s [3,4], which put into question different researchers' ability to identify the same worked materials. Despite many advances since the 1980s [see 5-7,8 for reviews], the identification of different worked materials is still considered an insufficiently developed area. This is a major setback for prehistorians, because residues are not always preserved, and knowing which materials were worked can make an enormous difference in interpreting area or site function, or in claims about style and cultural transmission, etc. The problem is manifold: not all polishes are equally easy to see and interpret, and not all types of data deliver the same answers. Some types of materials, such as cereals [9,10] or antler, bone, and ivory (ABI) have been identified consistently in blind tests or have been separated quantitatively in experimental settings with varying degrees of success (see [11-15]). Traces left by others, especially by soft and elastic materials, such as meat, tendons, etc., are less identifiable [16]. Several quantitative studies did report success in distinguishing target materials, but, even here, within-group similarities were not adequately explained. For example, Stevens et al.'s [15: Fig. 4] discriminant functions grouped antler with plant polishes. Moreover, some studies could not clearly distinguish wood [17] and hide polish [13, Table 4] from unworked areas. In one of the most recent studies, Ibanez et al. [12: 1188] report a similar overlap between some contact materials in their quantitative analysis as those noted by both other quantitative studies and also in blind tests [e.g., 18], despite studying one of the largest samples to date. Therefore, because the processes that lead to these differences and similarities in polish are not sufficiently well understood, we cannot understand why attempts to classify polishes produced by different materials fail or produce ambiguous results when they do. One obvious way to resolve this problem is to study the mechanical properties of prehistoric target (worked) materials and test their effects on wear development. Unfortunately, these materials are not commonly studied by tribologists, as they have no industrial applications. They are also more variable than industrial ones, as the available data show. For example, the varying amount of mineralization in bone, ivory, and antler causes mechanical properties to also vary among species [19], in some cases significantly so [20]). Further, these materials change their properties when they are wet vs. dry [21], or in very cold weather conditions [particularly ivory, see 22]. While some have claimed that this produces different traces on stone tools, why that might happen remains insufficiently explained. Given that it is now widely believed that abrasion is the main process by which stone tools are polished during use [23-25], it makes sense to test first the effect of hardness. Until now, systematic mechanical tests of archaeologically-relevant target materials have not been incorporated into lithic use-wear research. In particular, hardness tests were carried out on the stones themselves [26-29], but not on the target materials. For this reason, we decided to carry out a test of the target material hardnesses and to evaluate their role in abrading flint. 
 
 
 Method 
 
 1) The Experiment setup 
 To isolate the effect of material hardness while maintaining reasonable costs and effort, we opted for a tightly controlled protocol [30,31]. Flint bits (Baltic/morainic flint from Denmark with nano-crystalline non-oriented chalcedony texture [25]) were rubbed against dry bone, antler, and wood (beechwood and spruce) using a tribometer at room temperature. Five samples of each pair were produced to obtain a suitable number of samples for statistical study. The experiment was sequential; the flint samples were mounted on the tribometer (Nanovea T-50) (Fig 1) and used sequentially for 1, 3, and 5 hours. The tribometer variables were fixed to a load of 20N, a speed of 35 revolutions per minute and a straight back and forth motion. 
 
  
 Fig.1: Picture of the tribometer setup 
 
 
 
 2) Sample Preparation 
 The stone was first broken in small fragments using a hammer. The stone bits that had a flat natural surface on one side were selected. The opposite side was then adjusted to fit into the tribometer's clamp using a saw (Buehler, ISOMET TM, law speed saw) and then glued (Scotch Create Permanent Super Glue Liquid) to the clamp. The flat natural surface was rubbed against one of the worked materials selected. As polish develops unevenly on the sides of the sharp edge of a tool, this setup helps to create a sufficiently large polished area for carrying out microscopic analyses. The samples were cleaned before every documentation of wear. Because of the metallic nature of the clamp, a harsh cleaning protocol using acid and base was impossible and instead, a mild cleaning process was used. The samples were placed in individual plastic bags filled with a 10% neutral soap solution (Valconox, Luminox). They were then immersed into an ultrasonic bath for 15 minutes (Branson 5800, 40 kHz, at room temperature, 22C). Then they were rinsed with tap water and put in a distilled water bath for 5 minutes. Finally, samples were left to air-dry. 
 
 
 3) 3D Analysis 
 An optical profilometer (S-Neox, Sensofar Metrology, Barcelona, Spain) was used to collect surface topography measurements before the experimentation and then at each step of the experiment. At each step, 3 to 5 measurements of different areas were taken for each sample. Measurements were acquired with both a 20x objective (TU Plan Fluor EPI P; NA = 0.45; FoV = 872.68um 655.965um) and a 50x objective (TU Plan Fluor EPI P; NA = 0.80; FoV = 350.88um x 264.19um). Pictures were taken using the blue LED (530 nm) to obtain the highest resolution possible with our equipment. Only pictures with a surface measure higher than 98% were used for the analysis. The surface images were then analyzed using SensoMap (Standard 7.4). To extract surface parameters from the pictures, the filtering protocol presented in Calandra et al. [37] was used: (1) extraction of the topographic layer, (2) use of a Gaussian low-pass S-filter (S1 nesting index = 1.093 um for the 20x objective and 0.437 um for the 50x, end effects managed) to remove noise and keep the primary surface, (3) use of an F operator (polynomial of degree 3) to remove the form and keep the SF surface, i.e., texture, (4) use of a Gaussian high-pass L-filter (L nesting index = 327.980 um for the 20x objective and 131.200 um for the 50x, end effects managed) to filter out the waviness and keep the SL surface, i.e., roughness, and (5) setting threshold surface between 0.010 and 99.9% material ratio to remove the aberrant positive and negative spikes. It is important to note, however, that sometimes that threshold was readjusted depending on the persistence of the outliers. The cut-off values were calculated following ISO norms [38,39] recommendations; the L nesting index used was half the size of the shortest side (breadth) of the field of view, and the S1 nesting index was obtained by dividing the L nesting by 300 times. Four of the ISO25178 parameters were selected to perform the statistical analysis (Fig 3): arithmetic mean height (Sa), autocorrelation length (Sal), arithmetic mean peak curvature (Spc), and the upper material ratio (Smr1). These four parameters, relatively independent, provide an overall understanding of the surface textures [40]. 
 
  
 Fig. 3 From Martisius et al., 2018 (doi:  https://doi.org/10.1371/journal.pone.0206078.g004 ) 
 
 The statistical analyses were performed in the open-source software R (v. 3.5.2; R Core Team 2018) using the following packages: ggplot2 (v. 3.1.0; (Wickham, 2016), dplyr (v. 0.8.0.1; Wickham 2019), tidyr (v. 0.8.3; (Wickham, 2019b), ggpubr, MASS, and ggalt. Boxplots were produced to give a sense of the flint texture variation depending on the worked material hardness. Besides, an analysis of variance (ANOVA) and paired t-test were used to show which roughness parameters can help discriminate material types or hardness. 
 
 
 4) Hardness test 
 The hardness of each raw material was measured using a nano-indentation test. The samples were placed in resin using a SamplKwick Fast Cure Acrylic Kit 20-3560 (which contains SamplKwick Powder 20-3562 and SamplKwick Liquid 20-3564). The resin was prepared by a cold molding process done at room temperature by mixing two parts of 20-3562 SamplKwick Powder and one part of 20-3564 SamplKwick and blending thoroughly for 15-20 seconds. Then the mixture was poured into ring molds without delay. Before pouring the mix into the ring molds, we sprayed the cups with Mold release Spray (Buehler Mold Release Spray, 203050008). Finally, the surface of the cast was polished to obtain a smooth surface. The nanoindentation measurements were performed using a nanoindenter (Fig.3) (TI 950, Triboindenter, Hysitron, Minneapolis, MN) equipped with a diamond Berkovitch indenter. Prior to the experiment, the tip area function and the frame stiffness were calibrated using a fused silica standard. Nanoindentation measurements were conducted using the Oliver-Pharr or the quasi-static loading mode (Hay et al., 2010; Li et al., 2002; Oliver and Pharr, 1992) from which the reduced modulus ( \(Er\) ) and hardness were estimated. The initial unloading portion of the load-displacement curve represents purely elastic recovery. The slope of this unloading segment is a measure of the material contact stiffness (Oyen and Cook, 2009). The reduced Young's modulus ( \(E_{r}\) ) can be calculated by  \(E_{r} = \frac{1}{2}S\sqrt{\frac{\pi}{A_{max}}}\) , where  \(S\)  is the contact stiffness and  \(A_{max}\)  is the surface contact area at the maximum depth. The elastic modulus of the indented specimen, is computed using  \(\frac{1}{E_{r}}=\frac{1-\nu_{S}^2}{E_S}+\frac{1-\nu_{i}^2}{E_i}\) , where  \(\nu_S\)  is the Poisson's ratio of the indented specimen and  \(Ei\)  and  \(\nu_i\)  are the Young's modulus and Poisson's ratio, respectively, of the indenter. For a diamond Berkovitch indenter,  \(Ei\)  is equal to 1141  \(GPa\)  and  \(\nu_i\)  is equal to  \(0.07\)  (Oliver and Pharr, 2004). Since the Poisson's ratio of sedimentary rock ranges between  \(0.1 - 0.3\) , we have decided to report the  \(E_r\)  rather than estimate the  \(E_S\)  for modulus comparison. In addition, the hardness ( \(H\) ) can be calculated using the maximum load,  \(P_{max}\) , by depth. The elastic modulus of the indented specimen, is computed using  \(H=\frac{P_{max}}{A_c}\) , where  \(A_c\)  is the contact area of the indentation. These quasi-static measurements were performed with a 5 s load time and a 10 s dwell time at a maximum load of  \(3000 \mu N\)  for stones and  \(300 \mu N\)  for both bone and antler. 
 
  
 Fig.2 A: Schematic drawing showing the surface displacement during indentation; B: A typical indentation P-h curve where hc is the maximum true contact displacement during loading, hf is the final plastic depth from the P-h curves, and hm is the indentor displacement at peak load. 
 
 
 
 
 Results 
 
 1) Statistical results 
                  An analysis of variance (ANOVA) made on each parameter show that only the Sa (p < 0.001) parameter is the only one that is statistically different (Fig 5). Hence, Sa is the most suited of the four parameters tested to understand the worked material hardness's impact on polish formation. The results indicate that the worked material modifies the natural flint surface quantitatively, depending on its hardness. The softer a worked material is, the more the flint surface will be polished. 
 Paired t-tests with the Bonferroni correction results for the Sa parameter (see Table 2) show that it is possible to differentiate a flint piece before and after use only for the softest material, the spruce wood (p = 0.001). It is also possible to differentiate between the two wood types (p = 0.045). However, for antler, ivory, and bone, the paired t-tests show that the means before and after use were not significantly different. In term of duration of the action, the results were statistically significant after 3 hours of use. However, the significance increased after 5 hours of user in particular to distinguish between the two wood types. 
 
 Tab1 :paired t-test results with their significance 
 
 
 .y. 
 group1 
 group2 
 n1 
 n2 
 statistic 
 df 
 p 
 p.adj 
 p.adj.signif 
 
 
 
 
 Sa 
 antler 
 beechwood 
 10 
 5 
 3.7703086 
 11.719557 
 3.00e-03 
 0.0450000 
 * 
 
 
 Sa 
 antler 
 bone 
 10 
 10 
 0.5388624 
 17.567535 
 5.97e-01 
 1.0000000 
 ns 
 
 
 Sa 
 antler 
 ivory 
 10 
 5 
 0.6032733 
 12.638991 
 5.57e-01 
 1.0000000 
 ns 
 
 
 Sa 
 antler 
 raw 
 10 
 40 
 1.4770012 
 10.563733 
 1.69e-01 
 1.0000000 
 ns 
 
 
 Sa 
 antler 
 sprucewood 
 10 
 5 
 10.7461696 
 9.772127 
 1.00e-06 
 0.0000149 
 **** 
 
 
 Sa 
 beechwood 
 bone 
 5 
 10 
 -3.5174487 
 10.386906 
 5.00e-03 
 0.0750000 
 ns 
 
 
 Sa 
 beechwood 
 ivory 
 5 
 5 
 -3.5850431 
 7.819647 
 7.00e-03 
 0.1050000 
 ns 
 
 
 Sa 
 beechwood 
 raw 
 5 
 40 
 -3.7128110 
 4.824990 
 1.50e-02 
 0.2250000 
 ns 
 
 
 Sa 
 beechwood 
 sprucewood 
 5 
 5 
 6.1764210 
 4.414617 
 3.00e-03 
 0.0450000 
 * 
 
 
 Sa 
 bone 
 ivory 
 10 
 5 
 0.0525376 
 11.680601 
 9.59e-01 
 1.0000000 
 ns 
 
 
 Sa 
 bone 
 raw 
 10 
 40 
 0.9199148 
 11.167691 
 3.77e-01 
 1.0000000 
 ns 
 
 
 Sa 
 bone 
 sprucewood 
 10 
 5 
 11.6840847 
 10.044221 
 4.00e-07 
 0.0000054 
 **** 
 
 
 Sa 
 ivory 
 raw 
 5 
 40 
 0.9104150 
 5.136519 
 4.03e-01 
 1.0000000 
 ns 
 
 
 Sa 
 ivory 
 sprucewood 
 5 
 5 
 12.4557766 
 4.561824 
 1.07e-04 
 0.0016050 
 ** 
 
 
 Sa 
 raw 
 sprucewood 
 40 
 5 
 26.3346007 
 24.570355 
 0.00e+00 
 0.0000000 
 **** 
 
 
 ## 2) 
 microscope o 
 bservations 
  
  
  
  
  
  
  
 
 
 
 
  
 Fig.4: Polish development after 5 hours of use 
 
 Visually, it is noticeable that polish did not develop to the same extent on all the flint bits, even with the naked eye (Fig 4). According to the statistical results, the flint surface appears more polished when rubbed on spruce wood and beech wood. For the other worked material, the polish is sparser. 
 
 
 
 Discussion 
 The basic problem in explaining what causes stone tool wear is that most worked materials (such as skins, wood, bone, etc.) are softer than stone and cannot, in theory, abrade the stone tools themselves. However, given that stone tools are, in fact, abraded by contact with softer materials [23-25], why does this happen, and which factors related to the target material play the most significant role? Our counterintuitive result suggests that the abrasion 'paradox' must be explained by either the presence of hard grits within the worked material, the presence at the interface of stone particles broken off the tool edge, or a combination of both [48]. In the second case, the ability of softer materials to cause stone particles to break off via a combination of friction heat and mechanical dislodging is likely determined by their own structure and mechanical properties. Therefore, knowing exactly to which degree these properties are responsible for creating wear is crucial for being able to recognize different worked materials. The results obtained in this study confirm previous results stating that it is possible to differentiate between the softest material (woods) and harder ones (antler, bone, ivory) statistically. There also appears to be a statistical separation between the two types of wood, spruce and beech. However, using basic surface topography, it does not seem possible to distinguish among antler, bone, and ivory. These observations confirm previously reported difficulties in visually differentiating polish within this group, which are often reported together as ABI [49,50]. An unresolved issue is that, even after 5 hours of work in our setup, the polish was barely developed on harder materials, while it was almost completely flat in the wood samples. Hence, for ivory, bone, and antler, it was impossible to distinguish the before and after use surfaces based on changes in Sa. In our previous work scraping beech wood [51], quantifiable differences in Sa were only documented with higher loads, of 90N and 100N. However, here we were able to obtain changes in Sa for the same material, beech wood, even with the comparatively much lower load of 20N. The discrepancy can be attributed to the difference in the ability of that paper's instrument (using focus variation) to measure polish and/or, given other teams' successes using focus variation [13], to the lack of a standardized surface on which to measure it. However, it is also possible that higher loads or much longer working durations are required to make noticeable changes to the stone surface. This could be due to processes involving dislodging particles from the stone surface using friction heat, which remain to be documented experimentally. 
 
  
 Fig 5 Boxplots showing the evolution of flint roughness parameters Sa, Sal, Spc, and Smr1 after 1 hour, 3 hours and 5 hours of use on worked materials of various hardnesses 
 
 One reason that can explain these results could be the presence of more abrasive compounds in the softer worked material such as silicate. Residual powders from the track formed by the flint rubbing on the worked materials were collected and analyzed using scanning electron microscopy (SEM) and X-ray analysis (EDS) (Fig 6). Silica could not be found in the EDS framing the broad region of the samples, most likely because the other elements' noise was too heavy to enable the detection of other compounds or because the powdered silica are too small to be picked up by the EDS probe. However, we found that the interfacial powders from each raw material contained flint fragments of approximately 200??m. 
 
  
 Fig 6: X-ray microanalysis of the spruce wood powder 
 
 The filtering process in surface texture analysis is essential to obtain workable data while demanding physics knowledge behind the surface texture measurement. Additionally, the software has been conceived for engineering and industrial applications and the treatment of a predicted surface, not archaeological artifacts. However, there is no standardized method of filtering for stone tools or other archaeological material. The filtering options are numerous, and each use-wear study displays a different filtering method. This variability can affect the comparability of these controlled and quantified studies and would need to be standardized. In this case, the two softer materials happen to be wood, but silica content in temperate wood species is negligible [47], whereas the apatite contained in the bone matrix are softer than the quartz in flint. At the same time, because flint was found in track powders associated with all materials, it is possible that these particles contributed most to the abrasion, since they are hard enough to scratch the stone surface. However, we do not understand exactly how and why flint particles were broken off the stone surface in the first place. Although we tried to maintain a contact between two relatively flat surfaces to maximize contact area and minimize the variation in pressure distribution, because we opted for a natural raw surface, edge breakages were still possible. Moreover, it is possible that the particles we found with the aid of the EDS probe are too large to have really contributed significantly to polish development. 
 
 
 Conclusion 
 As discussed in another paper related to this experimental setup [25], polish is formed by abrasion of the flint surface. Consequently, we expect that harder worked material would be more effective in creating polish. However, our experiment shows that the softer the worked material is (i.e., the lower the arithmetical mean height (Sa)), the more the polish is developed. Given these results, it is imperative that other mechanical properties of materials typically expected to occur in prehistoric tasks be studied. These could include the materials' own surface roughness, elasticity, fracture toughness, the presence and brittleness of embedded grits, and others. In addition, longer work duration and higher loads (with different machines) can be tested to obtain more polish formation on stone samples used on harder materials. Additionally, for this pilot study, materials were used in the dry condition, but saturation with water and added lubrication (e.g., fat) should be included in future studies. 
 
 Acknowledgment: 
 Dr. Lukasz Witek, Z. hong Wang and Otto. Mullings from the Craniomaxillofacial Orthopaedic Biomaterials Regenerative Applications Lab, New York University College of Dentistry Department of Biomaterials and Biomimetics, for nanoidentation tests. 
 ``` 
 
 


 

 

 

 

 


 
 

 
 
