# Supplementary figures and images for: The effect of worked material hardness on stone tool wear
Source: PLoS One. 2022 Oct 20;17(10):e0276166. doi: 10.1371/journal.pone.0276166 (PMC9584531; doi:10.1371/journal.pone.0276166)

Str


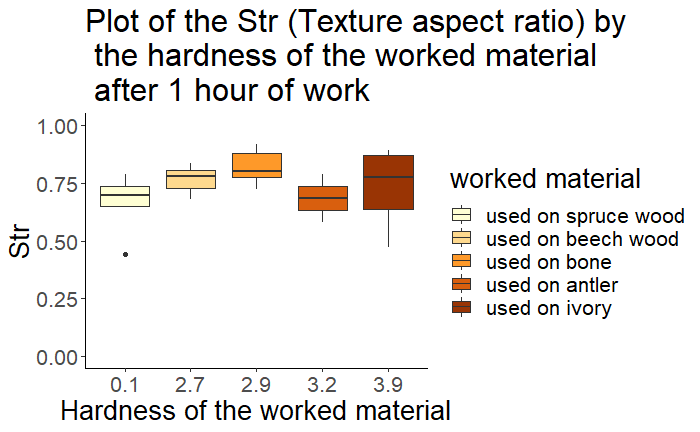


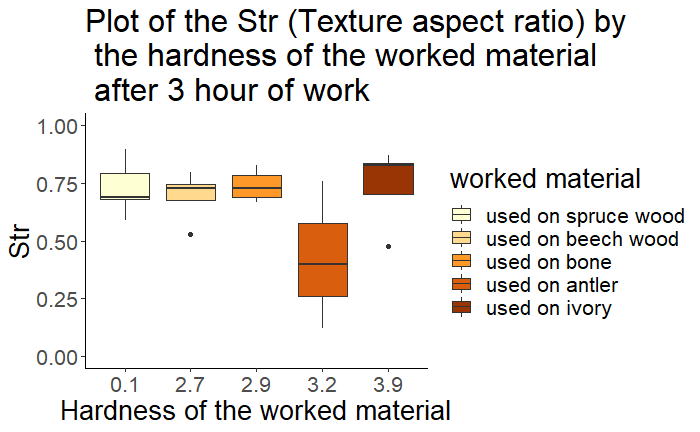


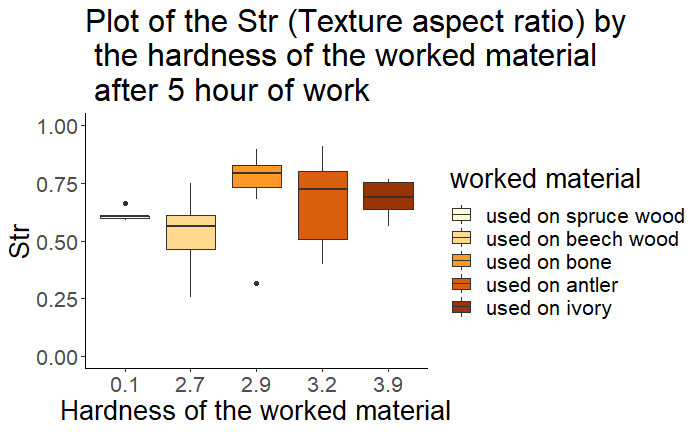


Std


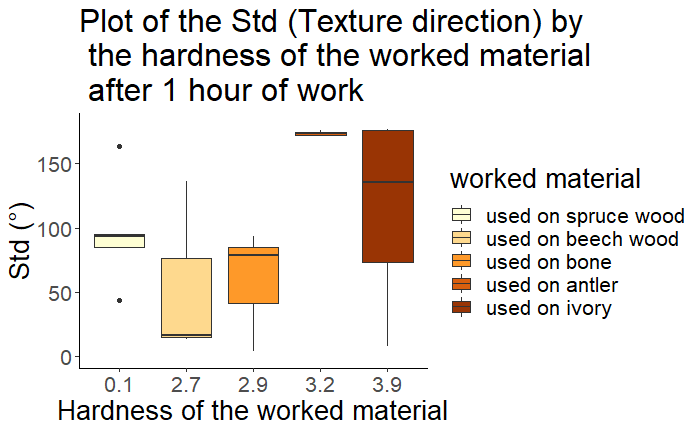


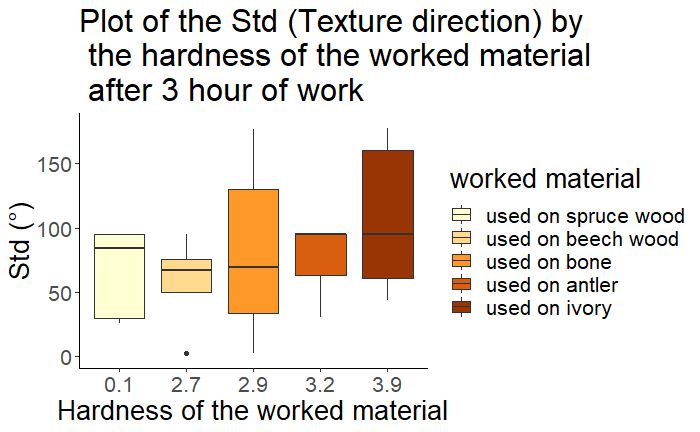


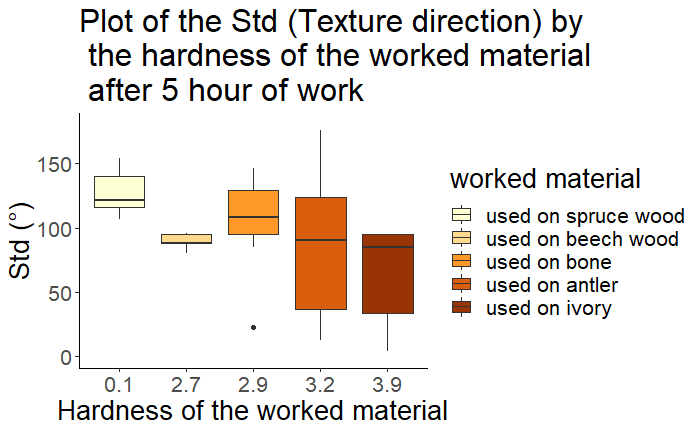


Spk


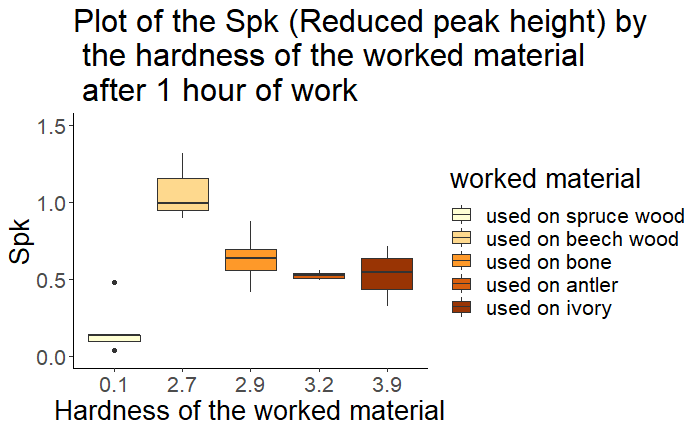


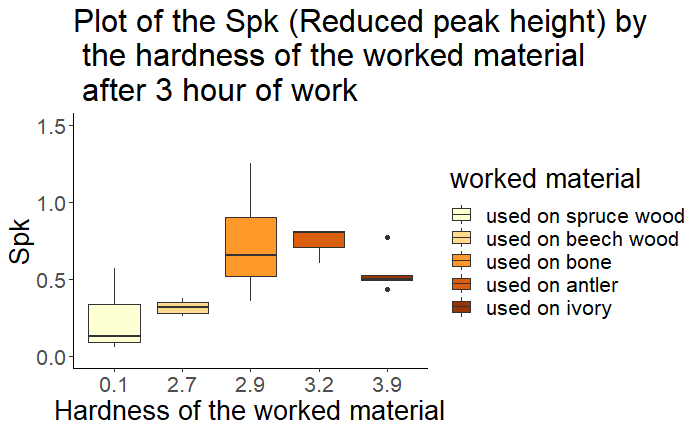


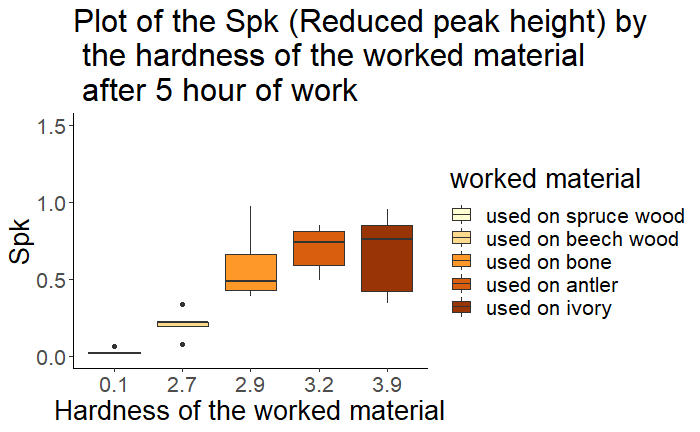


Sk


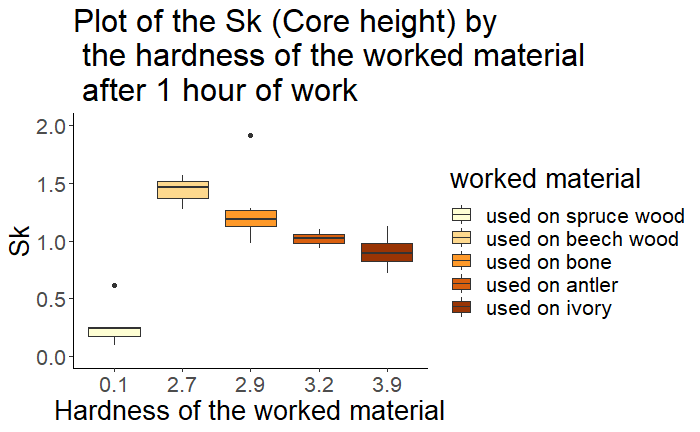


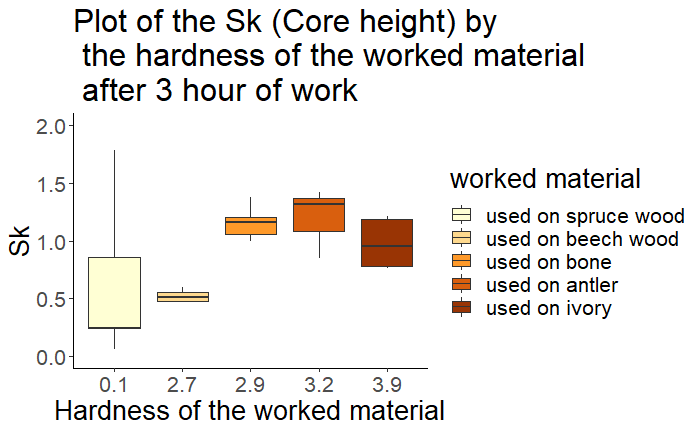


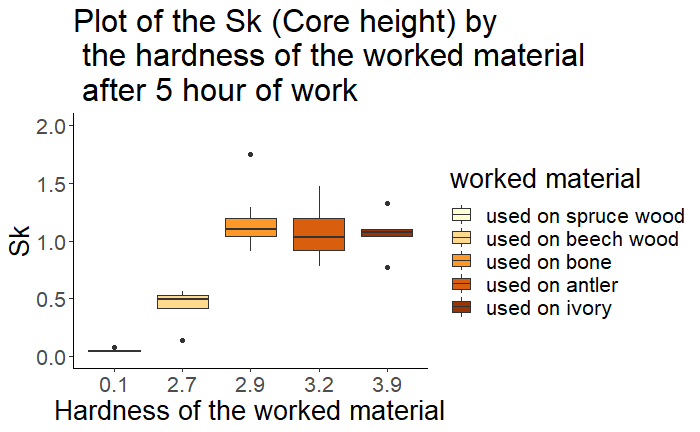

Supplement: S1 File — (ZIP) [file pone.0276166.s002.zip › Markdown Version/complementary parameters, results.docx]

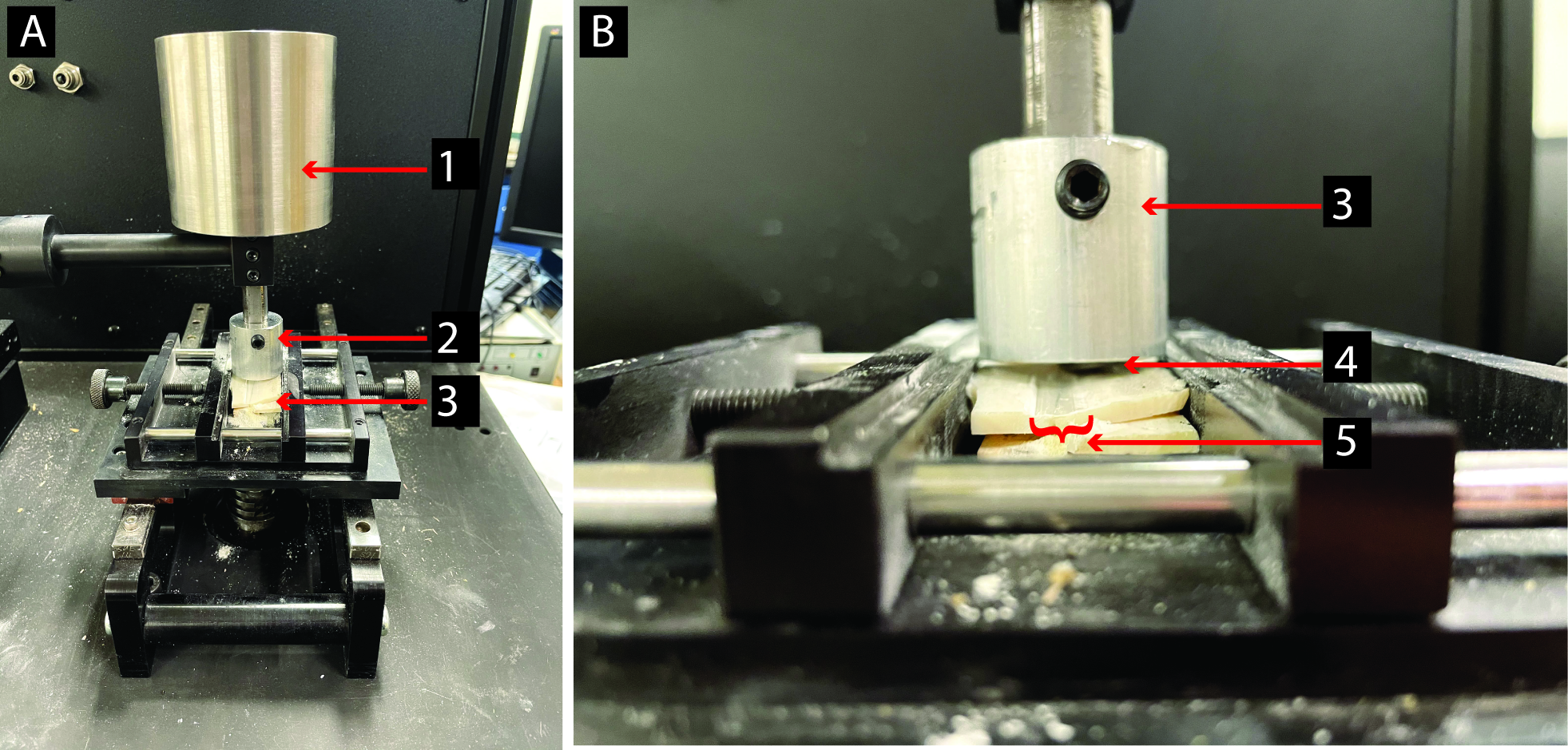

Supplement: S1 File — (ZIP) [file pone.0276166.s002.zip › Markdown Version/Fig 1.tif]

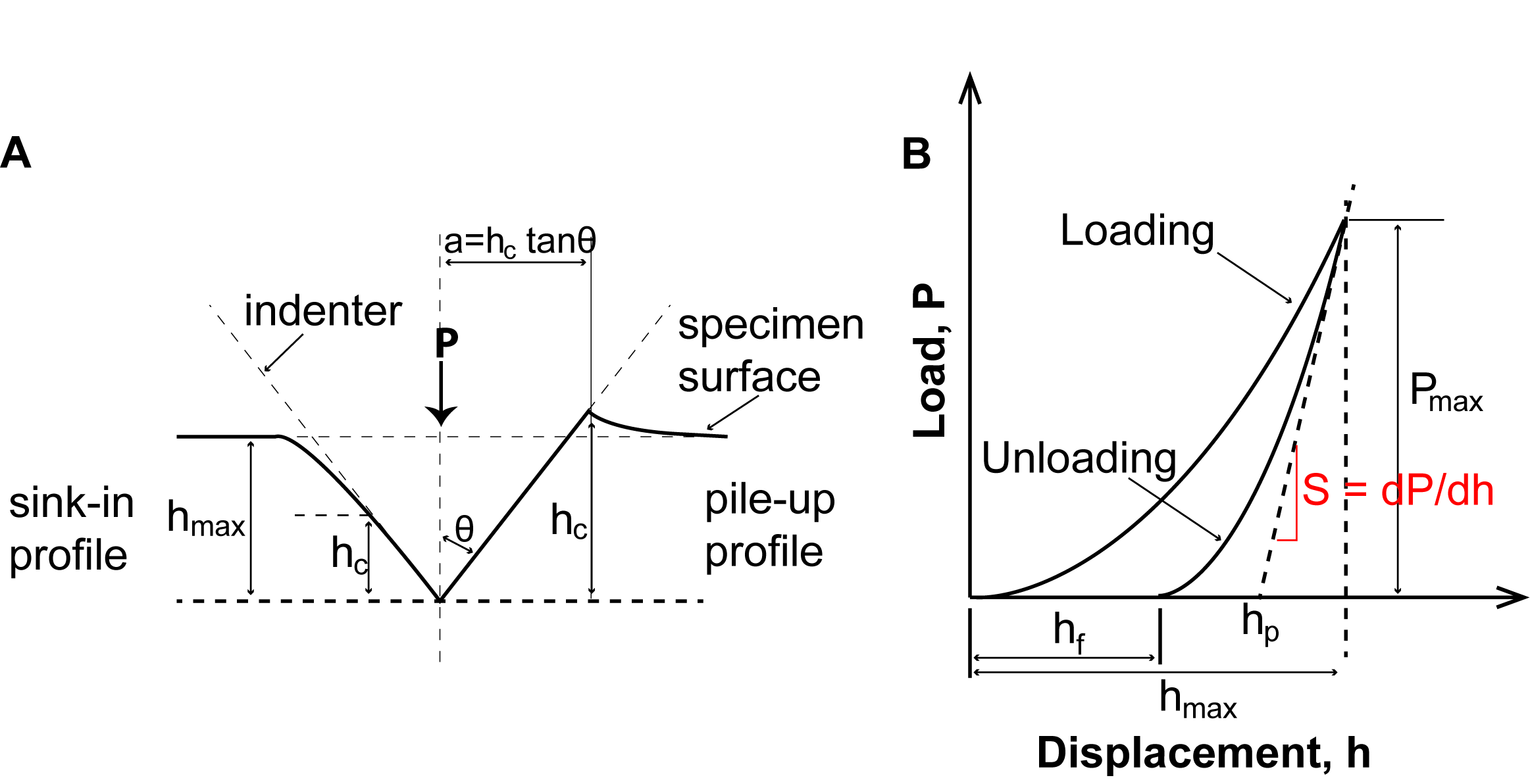

Supplement: S1 File — (ZIP) [file pone.0276166.s002.zip › Markdown Version/Fig 2.tif]

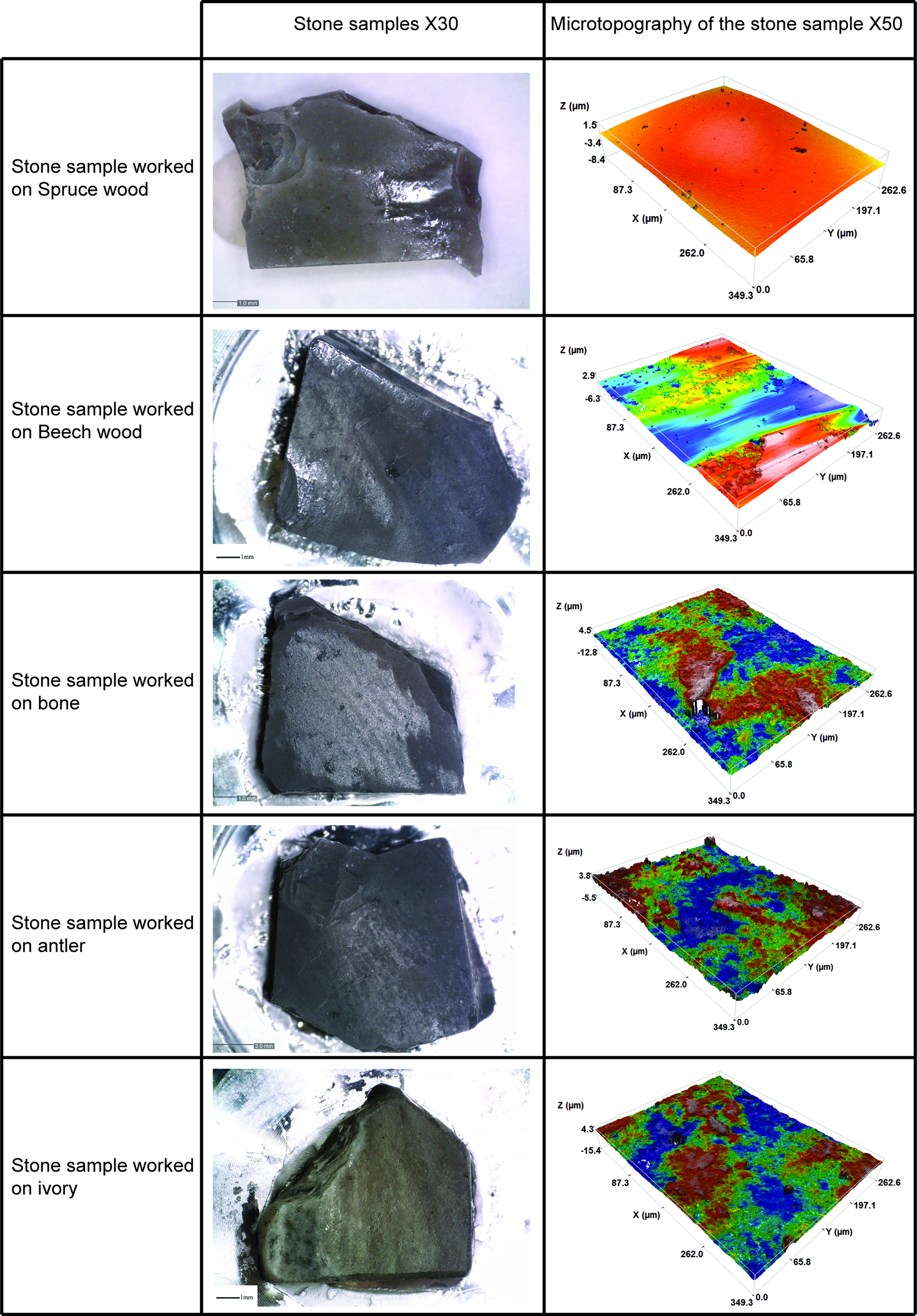

Supplement: S1 File — (ZIP) [file pone.0276166.s002.zip › Markdown Version/Fig 4.tif]

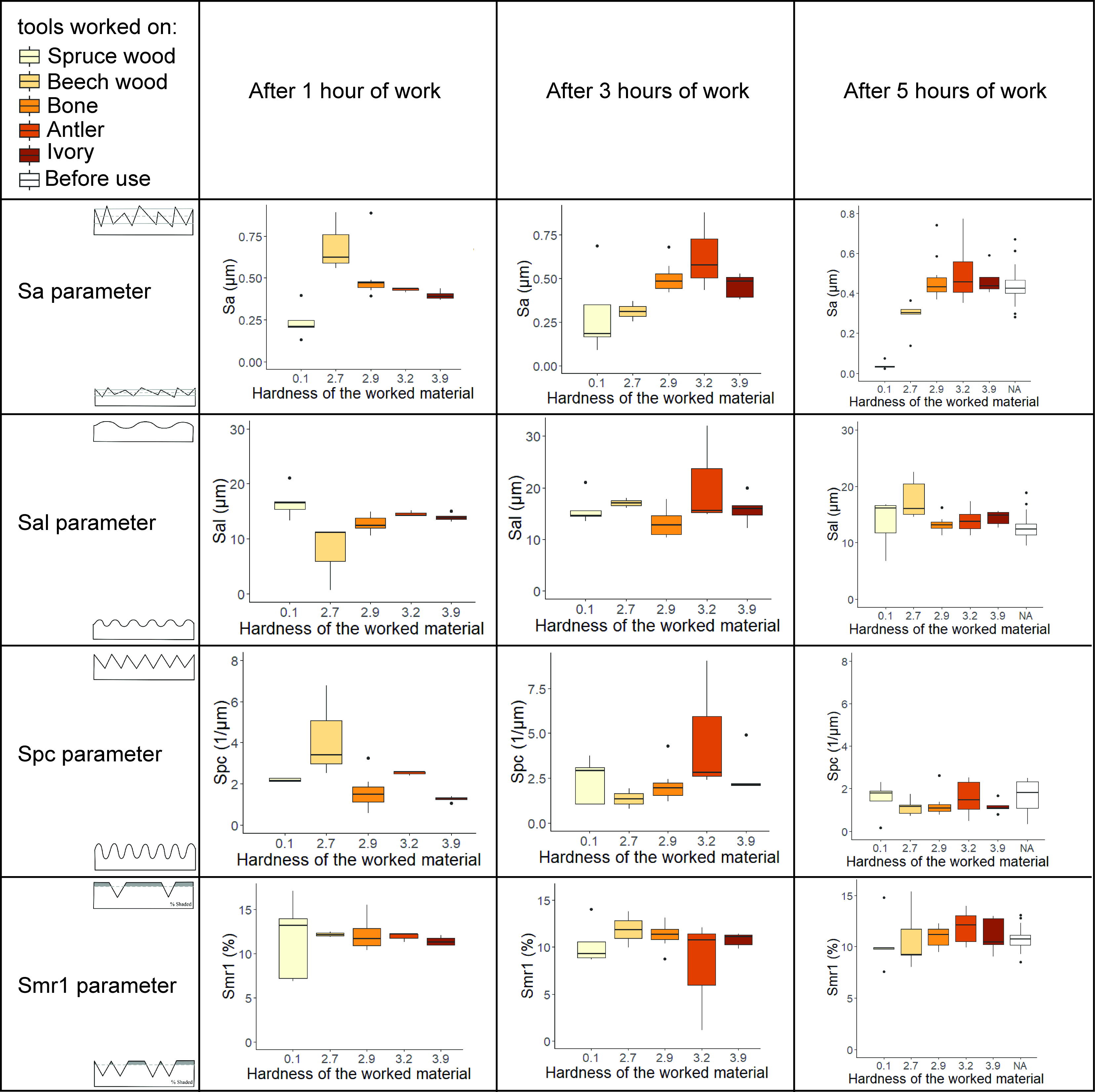

Supplement: S1 File — (ZIP) [file pone.0276166.s002.zip › Markdown Version/Fig 5.tif]

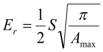

Supplement: S1 File — (ZIP) [file pone.0276166.s002.zip › Markdown Version/formula1.png]

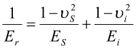

Supplement: S1 File — (ZIP) [file pone.0276166.s002.zip › Markdown Version/formula2.png]

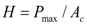

Supplement: S1 File — (ZIP) [file pone.0276166.s002.zip › Markdown Version/formula3.png]
